# Synthesis and Characterization of New Conjugated Azomethines End-Capped with Amino-thiophene-3,4-dicarboxylic Acid Diethyl Ester

**DOI:** 10.3390/ijms23158160

**Published:** 2022-07-24

**Authors:** Agnieszka Katarzyna Pająk, Sonia Kotowicz, Paweł Gnida, Jan Grzegorz Małecki, Agnieszka Ciemięga, Adam Łuczak, Jarosław Jung, Ewa Schab-Balcerzak

**Affiliations:** 1 Institute of Chemistry, University of Silesia, 9 Szkolna Str., 40-006 Katowice, Poland; agpajak@us.edu.pl (A.K.P.); jan.malecki@us.edu.pl (J.G.M.); 2 Centre of Polymer and Carbon Materials, Polish Academy of Sciences, 34 M. Curie-Sklodowska Str., 41-819 Zabrze, Poland; pgnida@cmpw-pan.edu.pl; 3 Institute of Chemical Engineering, Polish Academy of Sciences, 5 Bałtycka Str., 44-100 Gliwice, Poland; ciemiega@iich.gliwice.pl; 4 Department of Molecular Physics, Faculty of Chemistry, Lodz University of Technology, 116 Żeromskiego Str., 90-924 Lodz, Poland; adam.luczak@p.lodz.pl (A.Ł.); jaroslaw.jung@p.lodz.pl (J.J.)

**Keywords:** thiophene, azomethines, imines, 3,4-diethyl ester 2,5-diaminothiophene, thiophenoazomethines

## Abstract

A new series of thiophene-based azomethines differing in the core structure was synthesized. The effect of the central core structure in azomethines on the thermal, optical and electrochemical properties was investigated. The obtained compounds exhibited the ability to form a stable amorphous phase with a high glass transition temperature above 100 °C. They were electrochemically active and undergo oxidation and reduction processes. The highest occupied (HOMO) and the lowest unoccupied molecular (LUMO) orbitals were in the range of −3.86–−3.60 eV and −5.46–−5.17 eV, respectively, resulting in a very low energy band gap below 1.7 eV. Optical investigations were performed in the solvents with various polarity and in the solid state as a thin film deposited on a glass substrate. The synthesized imines absorbed radiation from 350 to 600 nm, depending on its structure and showed weak emission with a photoluminescence quantum yield below 2.5%. The photophysical investigations were supported by theoretical calculations using the density functional theory. The synthesized imines doped with lithium bis-(trifluoromethanesulfonyl)imide were examined as hole transporting materials (HTM) in hybrid inorganic-organic perovskite solar cells. It was found that both a volume of lithium salt and core imine structure significantly impact device performance. The best power conversion efficiency (PCE), being about 35–63% higher compared to other devices, exhibited cells based on the imine containing a core tiphenylamine unit.

## 1. Introduction

The compounds containing the imine bond (–N=CH–) known as imines, azomethines or a Schiff bases are a group of materials of interest to many fields of science [1,2,3,4,5]. Azomethines were tested as electrochromic materials and for applications in medicine and pharmacology [6,7,8,9], in optical computers [10,11] and also as agents preventing the corrosion of mild steel, zinc, aluminum and copper in an acid environment (corrosion inhibitors) [12,13]. Imines often exhibit electrical conductivity, and the properties of molecular glasses and can form morphologically stable layers, which are valuable properties in optoelectronic devices [14]. Schiff bases can act as solar filters and are investigated in photovoltaic cells as yes and active layers or components [15,16,17,18]. The imines were tested as hole transporting materials (HTM) in perovskite solar cells (PSCs). The hole transport layer (HTL) collects and transports holes from the perovskite layer, and may decrease the surface roughness of the perovskite and form better interfacial contact. Some of the azomethines were studied as HTM in PSC, allowed to reach power conversion efficiency (PCE) of even 14.37% [19]. On the other hand, the compounds containing a flat electron-rich (π-excess) thiophene ring with the possibility of modification and the formation of p-doped material constitute a significant structural element of the organic semiconductors [20]. The molecules with a thiophene ring(s) are p-type semiconductors, undergo a reversible electrochemical oxidation process, show a low oxidation potential and are characterized by a narrow energy band gap and high thermal stability [21,22]. Therefore, the thiophene derivatives are considered promising building blocks for most materials intended for organic electronics [23,24,25,26]. The appropriate structural modification, the number of thiophene rings and the degree of conjugation significantly impact the newly obtained compounds’ properties [23]. The thiophene-based materials were used as stable hole transport materials in hybrid PSCs [27]. Considering various thiophene derivatives, the compounds obtained from 2,5-diaminothiophene-3,4-dicarboxylic acid diethyl ester (DAT) are interesting as materials dedicated to organic electronics [23]. The condensation reaction of DAT with the various (di)aldehydes leads to the formation of (oligo)azomethines as promising materials for optoelectronic applications [14,21,22,28,29,30,31,32,33,34,35,36,37]. However, research related to the molecules prepared from 2,5-diaminothiophene-3,4-dicarboxylic acid diethyl ester is still valid, and further modifications of the chemical structure allow for the extension of the applicability range of these compounds [38,39,40,41]. Our research group also utilized the compound DAT as a precursor for conjugated azomethines [42,43,44]. The synthesized and reported symmetrical and unsymmetrical thiopheno azomethines from DAT have showed the beginning of the thermal decomposition above 200 °C, a promising low energy band gap (below 2 eV), emission from the S_2_ excited state and an ability to form an amorphous state. They were tested in the guest-host light emitting diodes (OLEDs) for the first time in our research group, and electroluminescence (EL) in the red spectral region were registered [42,43]. The most favorable EL results were obtained for the symmetrical imine with two N-phenylpyrrolidine substituents [43]. Unsymmetrical imines with one free amino group obtained from DAT were tested as transporting materials in hybrid solar cells (PSC) [44]. The devices with unsymmetrical imines showed higher power conversion efficiency than the reference device without an HTM layer. The most promising was a compound with a morpholine substituent. Attention should be paid to azomethines with a triphenylamine (TPA) core, due to the good charge-transport ability of the TPA unit and the low-cost synthesis of imines. The utilization of azomethine denoted as MS-2 with double 4-[N,Ndi(4-methoxyphenyl)amino]phenyl units as HTM in devices with the structure FTO/bl-TiO_2_/mp-TiO_2_/CH_3_NH_3_PbI_3_/MS-2/Au allowed for obtaining a PCE of 6.68% [45]. Salunke et al. [46,47] reported a series of imines with phenothiazine core end-capped with the TPA unit. The fabricated PSCs (ITO/SnO_2_/Cs_0_._05_MA_1−y_FA_y_PbI_3−x_Cl_x_/HTM/Au (or Ag)) showed PCE in the broad range of 9–14%. Petrus et al. [48,49] described a series of azomethines based on the TPA with different cores (i.a. EDOT, thiophene). The prepared PSCs (FTO/TiO_2_/CH_3_NH_3_PbI_3_/HTM/Au) showed a PCE of 0.2–14%. Bogdanowicz et al. [19] reported the symmetrical imine with two TPA units, which, applied in device FTO/TiO_2_/CH_3_NH_3_PbI_3_/bTAThDaz/Ag, resulted in registered PCE above 14%. The highest PCE=17% of device (ITO/HTM/CH_3_NH_3_PbI_3_/PC_61_BM/BCP/Ag Au) based on imines with TPA units was reported by Duan et al. [50]. The results become the motivation forfurther modification of the structure of thiophenoazomethines.

Our study focused on thiophenoazomethines and five new molecules with two free amino groups are presented. In this work, five new molecules with two free amino groups are presented as the results of our research group focused on thiophenoazomethines. New molecules were synthesized from DAT and dialdehydes such as a isophthalaldehyde, 4,4′-biphenyldicarboxaldehyde, 4,4′-diformyltriphenylamine and 2,2′-bitiophene-5,5′-dicarboxaldehyde, thieno[3,2-b]thiophene-2,5-dicarboxaldehyde. The effect of the compound core structure on thermal, optical and electrochemical properties was evaluated. Additionally, photophysical studies were supported by theoretical calculations using density functional theory (DFT). To estimate charge carriers mobility of thiophenoazomethine with triphenylamine, the Organic Field Effect Transistors (OFET) were made in a Top Gate Bottom Contacts (TGBC) configuration and used as a mobility indicator [51]. The template OFET were constructed with P3HT (*M*_w_ = 36,600) as a semiconductor film; in the indicator’s OFETs, the semiconductor film were replaced by thiophenoazomethine. The FET charge carriers mobility was calculated from I-V curves. Finally, the synthesized compounds were examined as HTM in hybrid photovoltaic cells.

## 2. Result and Discussion

### 2.1. Synthesis and Structural Characterization

**Figure 1:**
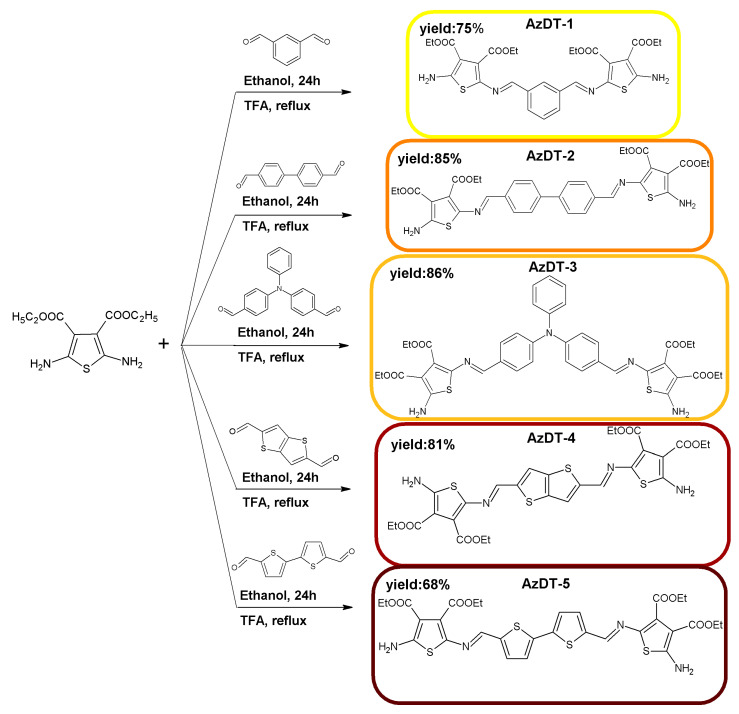
Synthesis route of thiophenoazomethines preparation.

Thiophenoazomethines(AzDTs) end-capped with donating amine group were obtained in the one-step condensation of the 2,5-diamino-thiophene-3,4-dicarboxylic acid diethyl ester with five dialdehydes (Figure 1 and Figure S1 in the ESI).

The compounds were synthesized in a mild reaction conditions with catalytic amounts of trifluoroacetic acid (TFA). Azomethines were obtained as a powders soluble in commercially available organic solvents. The ^1^H NMR, ^13^C NMR and FTIR investigations were performed to define the chemical structure of the synthesized thiophenoazomethines. In the^1^H NMR spectra of the compounds, the signal of the imine proton as a singlet was seen in the range of 8.02 (AzDT-3)–8.29 (AzDT-5) ppm (Figure S2 in the ESI). The amine (–NH_2_) proton signals as a singlet were seen at about 7.95 ppm. The lack of proton from the aldehyde unit was observed. The signals of the hydrogen atoms in the aromatic ring occurred in the typicalrange (7.06–7.87 ppm). The proton signals from –CH_3_ and –CH_2_– groups of the thiophene aliphatic chain were seen as quartets and triplets in the ranges of 4.14–4.32 ppm and 1.20–1.34 ppm (not symmetrical structures), respectively. Based on the infrared spectra, the absorption band of the imine unit from 1692 (AzDT-4) to 1649 cm^−1^ (AzDT-1 and AzDT-3) was detected and two absorption bands characteristic for the amine (–NH_2_) group at 3423–3465 cm^−1^ and 3308–3395 cm^−1^ were seen. The increase of conjugation was observed in compounds with a biphenyl (AzDT-2) and a bithiophene core (AzDT-5) with respect to compounds with a phenyl(AzDT-1) and a thieno[3,2-b]thiophenecentral unit (AzNT-4). The elongation of the π-conjugation system was confirmed by the shift of the position of imine proton signals towards higher values in the ^1^HNMR spectra and the shift of the absorption band of the imine group towards lower cm^−1^ values in the FTIR spectra. Additionally, the chemical structure of the prepared compounds was confirmed by HRMS. Elemental analysis results were in good agreement with theoretical, which indicates the purity of the synthesized thiophenoazomethines.

The thermal behavior of the AZDTs molecules was analyzed based on differential scanning calorimetry (DSC) measurements. The data obtained from DSC investigations are collected in Table 1, and exemplary thermograms are presented in Figure S3 in Supplementary Information (ESI).

In the DSC thermograms registered under the first heating scan, two endothermic peaks were observed, expect for the compound with a triphenylamine core (AzDT-3). The first endotherm corresponds to crystal to crystal transition (T_m_ ≈ 118 °C), whereas the second is a melting one, which indicates that AzDTs molecules were obtained as crystalline compounds. The second heating scan (after rapid cooling) revealed a glass transition temperature (T_g_) in the range of 101–194 °C, meaning the investigated molecules showed the ability to transform from crystalline into the amorphous state. No melting temperature during further heating above T_g_ was seen, except for the AzDT-2 with the biphenyl core. It means that AzDTs compounds form stable molecular glasses.

### 2.2. Electrochemical Investigations

**Figure 2:**
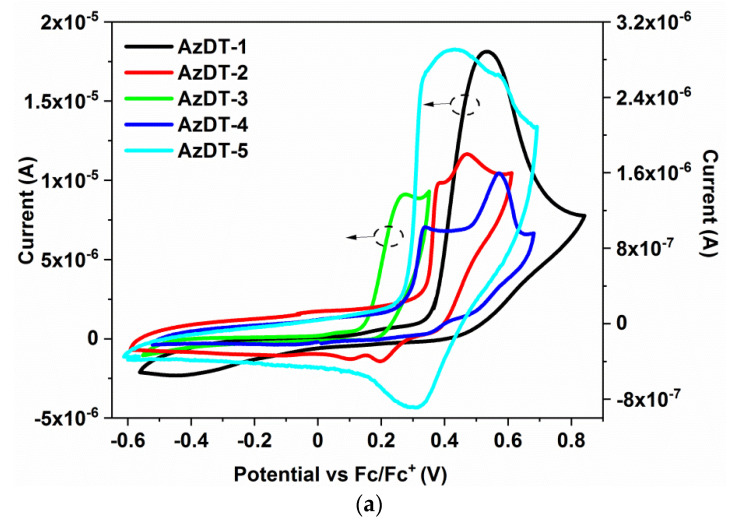

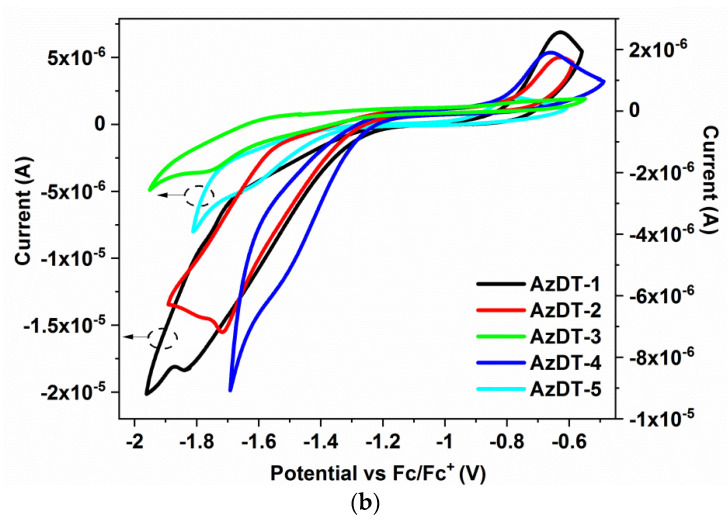
The voltammograms of the (**a**) oxidation and (**b**) first reduction processes (Pt, v = 0.1 V/s, 0.1 mol/dm^3^ Bu_4_NPF_6_ in CH_2_Cl_2_ with 10^−3^ mol/dm^3^ concentration of AzDT).

Cyclic voltammetry (CV) and differential pulse voltammetry (DPV) were used for the electrochemical investigations. The measurements were performed in 0.1 M Bu_4_NPF_6_ electrolyte in dichloromethane with 10^−3^ mol/dm^3^ concentration of AzDTs. The ionization potentials (IP) and electron affinities (EA) were estimated based on the onset potentials from the oxidation and reduction processes (E_ox(onset)_^1^,E_red(onset)_^1^). The data from CV and DPV are provided in Table 2 and the cyclic voltammograms are presented in Figure 2.

The investigated azomethines were electrochemically active and the oxidation and reduction processes were registered. The first reduction and oxidation process were irreversible [28], except for molecule AzDT-5 with a bitiophene core where the quasi-reversible process of oxidation was seen (ΔE = 110 mV). The two or three reduction processes (Table S1 in the ESI) were associated with the reduction of the acceptor part of the molecules with radicals formation and the imine bond, as was reported previously [43,44]. In the case of oxidation, more complex voltammograms were recorded, which may be related to the presence of the electron donating elements in the AzDTs’ molecules. Based on the oxidation potentials, it can be concluded that the compound AzDT-3 with a TPA core has greater capabilities to donate electrons because the oxidation process took place at a lower potential. Moreover, the compound AzDT-3 showed multistep oxidation processes (cf. Figure S4 in the ESI) in both CV and DPV measurements. The one para position in the TPA core is free and the formed radical cation can dimerize [52,53].

The reduction of the formed dication was seen in the reverse voltammetric sweep and in the second scan, and a new peak at 0.1 V was observed [53]. It should be noted that in order to see this behavior, the potential must be higher than 0.6 V vs. Fc/Fc^+^ (Figure S4 in the ESI). The other scans did not reveal the polymerization of the investigated molecules on the Pt electrode. The EA and IP, closely related to LUMO and HOMO levels, were obtained in the range of −3.60–−3.86 eV and −5.17–−5.46 eV, respectively (Table 2). The presence of the bithiophene (AzDT-5) and thieno[3,2-b]thiophene (AzDT-4) in the cores impacts on the IP and EA value, lowering the EA and increasing the IP and finally reducing the electrochemical energy band gap (E_g_) calculated as a difference between IP and EA. The energy band gap was below 1.7 eV and was dependent on the core structure (AzDT-1, 2, 3 > AzDT-4, 5), as mentioned above.

### 2.3. Theoretical Calculations

Theoretical calculations were performed with the use of the density functional theory (DFT) and were carried out using the Gaussian09 program on the B3LYP/6–311g++ level. Molecular geometry of the singlet ground and S_1_, S_2_, T_1_, T_2_, T_3_ excited states of the compounds were optimized in the gas phase (ground state) and electronic structures, and electronic transitions and excited states were calculated with use of the Polarizable Continuum Model (PCM) in dichloromethane for comparison of HOMO and LUMO energies with electrochemical data and chlorobenzene (excited states) as solvents. The optimized geometries of the compounds are depicted in Figure S6 in the ESI.

Comparing the energies of HOMOs and LUMOs determined on the basis of electrochemical data (cf. Table 2) with theoretically calculated values, it can be noticed that the calculated HOMO energies correspond with the experimental values of IP determined from CV measurements. Calculated LUMO energies were overestimated but the calculated values of the HOMO and LUMO energies were used only for consistency with geometry optimization. For a more detailed description of the molecular orbitals, the contribution of molecule parts, i.e., central fragment,–N=CH–and thiophene-3,4-dicarboxylic acid diethyl ester moieties to a molecular orbital, was calculated. The obtained DOS diagrams are presented in Figure S7 in the ESI, and the composition of selected molecular orbitals are gathered in Table S2 (contours of HOMO and LUMO are presented in Figure S8). HOMO comprises the conjugated bonds in the central molecule part, and the imine bond with the dominant share of the DAT fragment. LUMO is mainly localized in the central molecule part with the azomethine fragment. HOMO-1 and HOMO in AzDT-1 and AzDT-2 are degenerate with an energy difference of 90 meV and 200 meV, respectively. The presence of two donor moieties should lead to a degeneracy of the frontier orbitals, but in other compounds (AzDT-3, 4 and 5), the HOMO-1/HOMO energy differences are higher (310–480 mV), which is associated with changes in the acceptor fragment of the molecule. The influence of the donor on the LUMO levels is significantly attenuated (Figure S8 and Table S2 in the ESI).

The excitation wavelengths resulting in emission (*vide infra*) correspond to H-1/HOMO→LUMO/L+1 transitions (Table S3 in the ESI) andhave a mixed intra molecular charge transfer/locally-excited (ICT/LE) nature. Based on the data in Table S3, the charge transfer process takes place between the moieties and central molecule part including imine linkers. These compounds exhibit photoluminescence with low quantum yields (cf. Table 3) and the TD-DFT method was used to optimize the S_1_, S_2_ and T_1_, T_2_ T_3_ excited states of the AzDT-4 and AzDT-5 compounds in chlorobenzene as a solvent (in the case of the others compounds, the optimization of the excited states were not convergent). The emission spectra of AzDT-4 and AzDT-5 calculated for S_1_ show peaks in 548 and 551 nm, respectively. The transitions have a ^1^π→π^∗^ character and the contribution to the bands mainly comes from LUMO→H-1 (AzDT-4) and L+1→HOMO transitions (AzDT-5). Since the geometries of S_0_ and S_1_ states are similar (Table S4 in the ESI), the Stokes shifts are small (cf. Table 3). The geometries of the triplet states are also similar to the ground state but T_1_ presents a larger stabilization compared to S_1_ and a much lower energy compared to the ground state. Whereas the energy vertical emissions from S_1_ to S_0_ is close to 1.91 eV (649 nm, corresponding to the lower energy emission band cf. Table 3), the vertical emission from T_1_ is only 0.42 eV.

**Figure 3:**
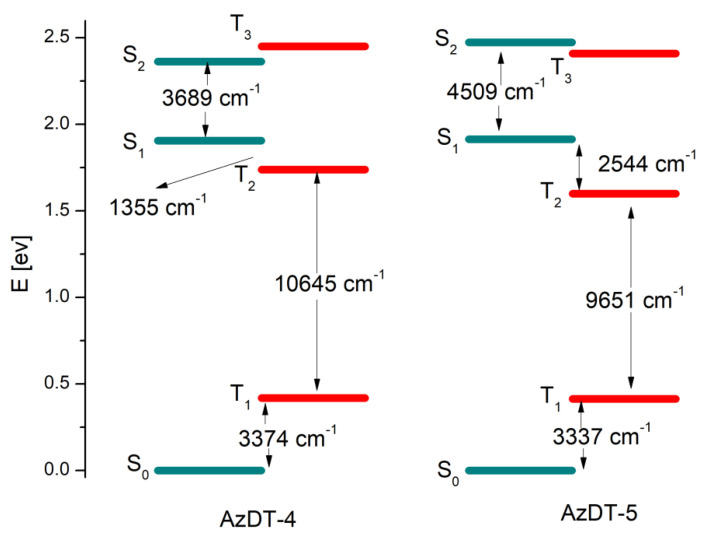
Low-lying energy states of AzDT-4 and AzDT-5 molecules.

The energy difference between the S_1_ and T_2_ state equal to 1335 cm^−1^ in AzDT-4 and 2544 cm^−1^ in the case of AzDT-5 indicates that the conversion process can easily take place (Figure 3). On the other hand, energy differences between T_2_ and T_1_ states is higher (~10,000 cm^−1^); therefore, the S_1_→S_0_ emission is observed, although non-radiative excitation energy dissipation processes related to internal conversion significantly reduce the fluorescence emission (see Section 2.4).

### 2.4. Photophysical Properties

**Figure 4:**
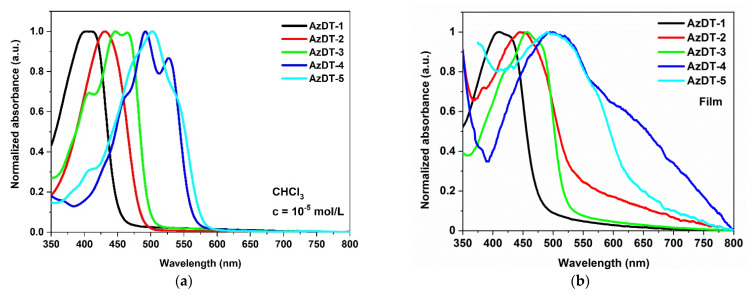
The UV-Visspectra of AzDTs in chloroform (**a**) and in (**b**) thin film.

The photophysical properties of AzDTs were investigated using UV-vis and photoluminescence spectroscopies. The UV-Vis spectra were recorded in the four solvents differing in polarity: chloroform (CHCl_3_, ε = 4.81), chlorobenzene (C_6_H_5_Cl, ε = 5.62), dichloromethane (CH_2_Cl_2_, ε = 10.66) and acetonitrile (C_2_H_3_N, ε = 37.50) in concentration c= 10^−5^ mol/dm^3^ and as a films prepared on the glass substrates. The electronic spectra are presented in Figure 4 (Figure S9 in the ESI) and data are collected in Table 3.

The imines in a solution absorbed the radiation with the maximum absorption band (λ_max_) located between 242 and 533 nm (2.22–5.12 eV; Table 3.). The absorption at higher energy ranges (3.82–5.12 eV) can be assigned to π→π* transitions [39,44]. The dominating absorption bands were localized at the lower energies (2.33–3.09 eV) and were shifted towards longer wavelengths depending on the core structure: phenyl < biphenyl < triphenylamine < bitiophene < thieno[3,2-b]thiophene (Figure 4). There were no significant differences in the *λ_max_* position registered in various solvents (Δ*λ_max_*= 2–12 nm; Figure S9 in the ESI). In the films, the maximum of the absorption bandwas very similar to *λ_max_* in the solutions (cf. Table 3 and Figure S9 in the ESI); however, the film of AzDT-3 (with TPA core) prepared from a chloroform solution showed a 10 nm red shift of the *λ_max_* compared to the solution. The broad absorption spectrum is presented in Figure S9 in the ESI, and was recorded for imines with a bitiophene (AzDT-5) and a thieno[3,2-b]thiophene (AzDT-4) core with the *λ_max_* located at the highest absorption coefficient in the solutions.

**Figure 5:**
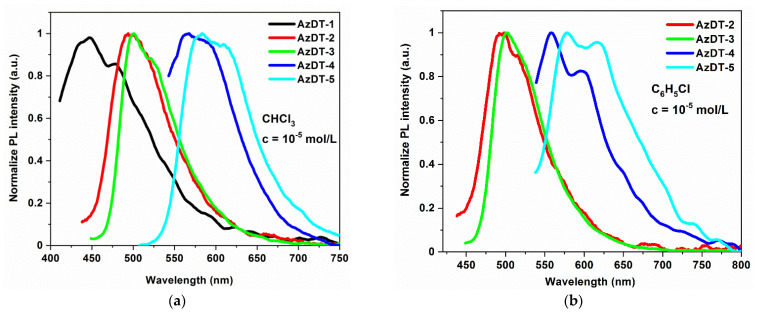
The emission spectra (PL) (**a**) in chloroform and (**b**) in chlorobenzene solution (λ_ex_taken from the UV-Vis spectra, presented in Table 3).

The presented molecules showed weak light emission in the solutions, and the PL quantum yield (ϕ) was below 2.5% and was none-emissive in the solid state. Such behavior was also reported in our previous publications for unsymmetrical and symmetricalthiophene-based azomethines [43,44]. The excited states are deactivated in a non-radiative way, which may be related to the presence of a heavy atom (sulfur) and internal conversion. In the solutions, the PL spectra were shifted towards the longer wavelengths depending on the core structure, as in the case of the absorption spectra (Figure 5).

The weak emission spectra with the one emission band were registered in the blue (AzDT-1, 2, 3), green (AzDT-2 in C_2_H_3_N, AzDT-4) and yellow to orange (AzDT-4 in C_2_H_3_N and C_6_H_5_Cl, AzDT-5) range of light. The maximum of the PL band *(λ_em_*) was bathochromic and shifted as the polarity of solvent increased (Table 3). In the case of AzDT-4 and AzDT-5 in the chlorobenzene solution, the vibrionic structure of the emission band was seen (Figure 5b). It was found that the excitation wavelength (*λ_ex_*) did not effect on the *λ_em_* position, according to the Kasha’s rule [54].

### 2.5. Photovoltaic Study

**Figure 6:**
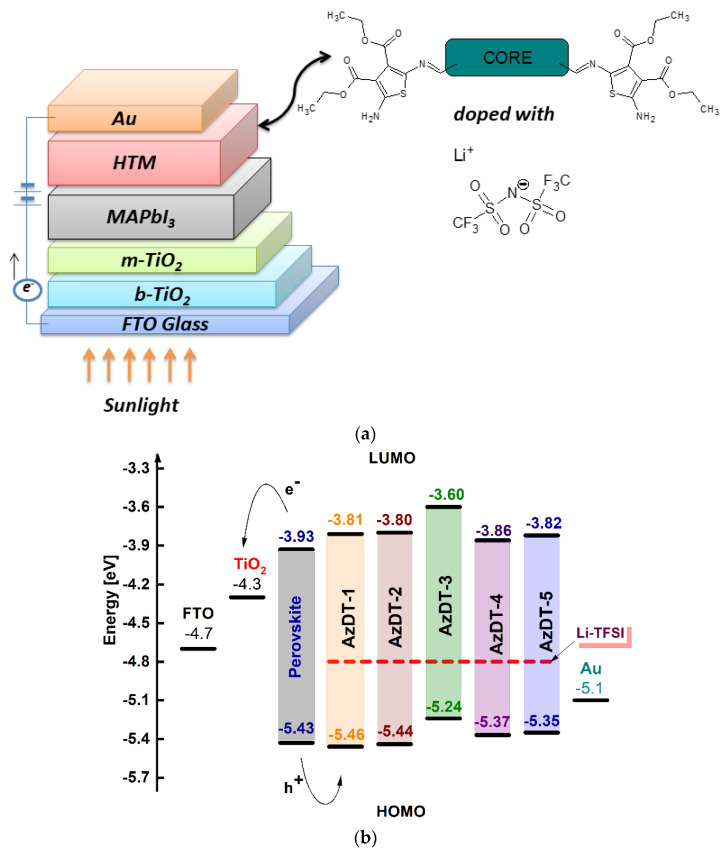
(**a**) The sandwich hybrid solar cell structure and (**b**) the energy level diagram of the cell components.

Considering the requirements for HTM, the energy of the HOMO of the HTM should be close to the energy of valance band of the perovskite for proper hole transport and the energy of the LUMO of the HTM should be higher than the energy of conductive band of the perovskite to block the electron flow to the Au electrode. The synthesized imines were tested asthe hole transporting materials in the non-encapsulated hybrid inorganic-organic perovskite solar cells (Figure 6b). The devices without the HTM layer (FTO/b-TiO_2_/m-TiO_2_/perovskite/Au) and with a Spiro-OMeTAD as the HTM were also fabricated. The PSC structure with the HTM layer is presented in Figure 6. The two-step method was applied for the perovskite layer (MAPbI_3_) preparation, which is described in Supplementary Information. To improve the efficiency of the cells, azomethines were doped with a different volume of lithium bis-(trifluoromethanesulfonyl)imide (Li-TFSI) with 4-tert-butyl pyridine (tBP) V_tBP_ = 28.8 μL, the common *p*-dopant (to “extract” the electrons from the HTM donor molecule; V_Li-TFSI_ = 8.75, 17.50 and 35.00 μL) [55,56].

**Figure 7:**
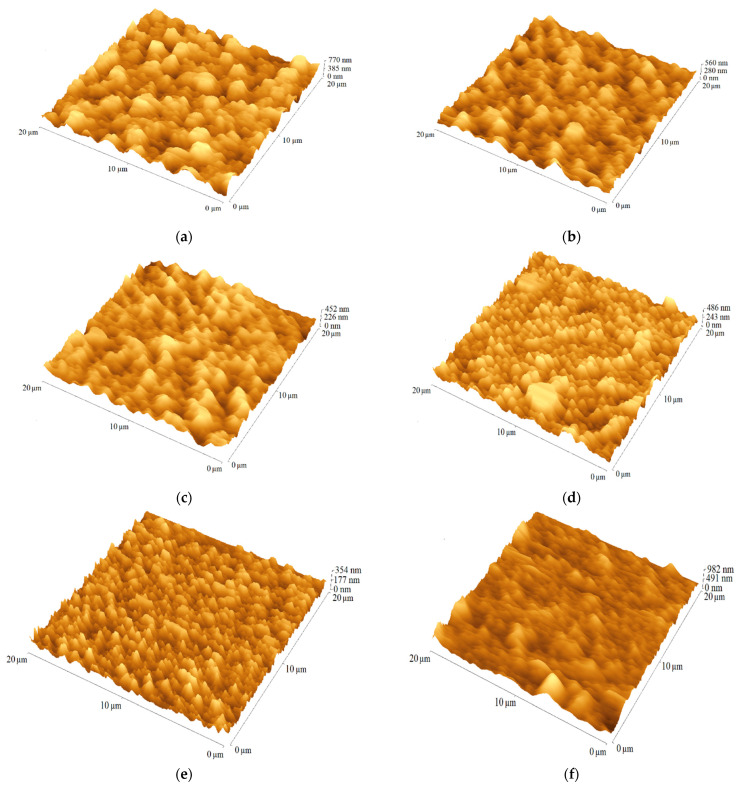

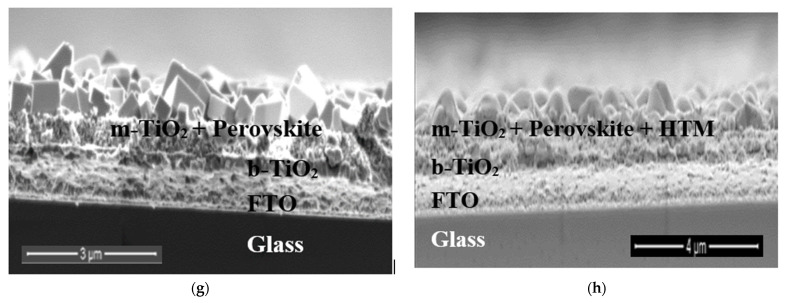
AFM images (20 µm × 20 µm) of (**a**) FTO/b-TiO_2_/m-TiO_2_/perovskite, (**b**) FTO/b-TiO_2_/m-TiO_2_/perovskite/AzDT-1, (**c**) FTO/b-TiO_2_/m-TiO_2_/perovskite/AzDT-2, (**d**) FTO/b-TiO_2_/m-TiO_2_/perovskite/AzDT-3, (**e**) FTO/b-TiO_2_/m-TiO_2_/perovskite/AzDT-4, (**f**) FTO/b-TiO_2_/m-TiO_2_/perovskite/AzDT-5 and the SEM cross-section images of (**g**) FTO/b-TiO_2_/m-TiO_2_/perovskite and (**h**) FTO/b-TiO_2_/m-TiO_2_/perovskite/AzDT-4.

The atomic force microscope (AFM) was used to estimate the quality of the layers based on the root-mean-square (RMS) parameter (cf. Table S5). The AFM micrograms of the tested surfaces are shown in Figure 7. Moreover, the scanning electron microscope (SEM) was utilized to register a cross-section images of the FTO/b-TiO_2_/m-TiO_2_/perovskite/AzDT-4 and the reference cell without HTM. The surface roughness of the oxide semiconductor (TiO_2_) was determinate. The RMS of the TiO_2_ mesoporous layer was about 20 nm, indicating a relatively planar structure. The deposition of the perovskite crystals resulted in a significant increase of the RMS value to about 130 nm (Figure 7a,g,h and Figure S11a). The presence of a hole transporting layer on the top of the perovskite decreased the surface roughness to 75–90 nm (cf. Table S5 and Figure S11b). The layer of imine with a biphenyl core (AzDT-2) showed the smoothest surface (RMS = 75 nm). The well-formed structure of the perovskite before and after HTM deposition is shown in the SEM images (Figure 7g,h).

**Figure 8:**
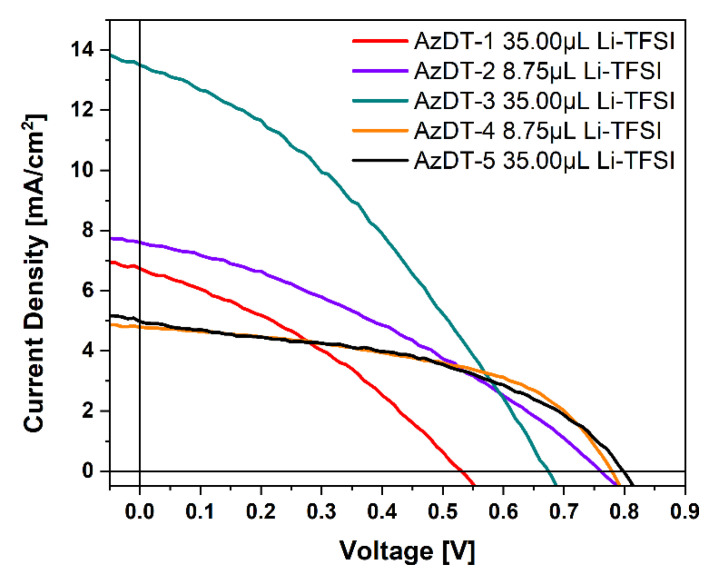
The current-voltage (I–V) characteristics for selected hybrid perovskite solar cells devices.

The photovoltaic parameters such as *J_sc_*—density of short-circuit current, *V_oc_*—open-circuit voltage, FF—fill factor, and PCE—power conversion efficiency estimated from current-voltage (I-V) characteristics are summarized in Table 4 and in Table S6 in the ESI. I-V graphs for the selected devices are collected in Figure 8.

The prepared solar cells with a HTM layer exhibited higher power conversion efficiency (PCE) than device without a hole transporting compound (Figure S12); however, for the solar cells with AzDT-2 and AzDT-5 (V_Li-TFSI_ = 35 μL), this difference was inconsiderable (Table S6). The highest *J_sc_* (density of short-circuit current, *J_sc_* = 13.50 mA/cm^2^) for the device structure FTO/b-TiO_2_/m-TiO_2_/perovskite/AzDT-3/Au (V_Li-TFSI_ = 8.75 μL) was achieved and resulted in the highest PCE (3.64%). However, such a value is lower compared to the PCSs based on the typical HTM, as is seen with Spiro-OMeTAD (Table 4). At the same time, the obtained efficiency (PCE = 5.05%) of cells with Spiro-OMeTAD is not high compared to the recorded results. Performing optimization of cell preparation would improve the PV performance of cells. However, the optimization was not the aim of this work. It should be noticed that the devices are prepared and measured in ordinary laboratory conditions without the use of appropriate, efficient systems to eliminate moisture and oxygen.

The charge carrier mobility was estimated for imine AzDT-3, which applied as a HTM gave the best results. The charge carrier mobility was measured based on the transfer current-voltage characteristic of the prototype OFET devices (Figure S13) [57,58,59]. The p-type characteristics were received and the hole mobility at 1.0 × 10^−4^ cm^2^/V·s were estimated in the saturation regime of OFET with an active layer ofAzDT-3. The increasing of lithium salt (8.75 μL < 17.50 μL < 35.00 μL) did not guarantee an increase to the power conversion efficiency of the investigated devices [60]. The radical cation formation affects the PCE. The dopant concentration is important and controlling the radical formation process is difficult [61,62,63].

## 3. Methods and Materials

Information concerning the characterization methods, film and device preparations with DFT calculations are available in Supplementary Materials.

### 3.1. Materials

Isophthalaldehyde and 4,4′-biphenyldicarboxaldehyde were purchased from Acros Organics and 2,2′-bitiophene-5,5′-dicarboxaldehyde from TCI. In addition, 4,4′-Diformyltriphenylamine, thieno[3,2-b]thiophene-2,5-dicarboxaldehyde, trifluoroacetic acid (TFA), activated charcoal, KBr, Bu_4_NPF_6_ and solvents were purchased from Sigma Aldrich (Merck, Rahway, NJ, USA). The materials used for perovskite solar cells were surfactant, fluorine doped tin oxide coated glass slides (FTOs, 7 Ω/sq, Sigma-Aldrich, St. Louis, MO, USA), ethanol (EtOH, POCH), hydrochloric acid (HCl, CHEMPUR), tetraethyl orthotitanate ((C_2_H_5_O)_4_Ti, Merck), paste Ti-Nanoxide T/SP (Solaronix), anhydrous N,N-dimethylformamide (DMF, Sigma-Aldrich), isopropanol (IPA, POCH), lead iodide (PbI_2_, Sigma-Aldrich), methylammonium iodide (MAI, Solaronix) and chlorobenzene (C_6_H_5_Cl, POCH). Additionaly, 4-Tert-butyl pyridine (TBP) and lithium bis(trifluoromethanesulfonyl)imide (Li-TFSI) were purchased from Sigma-Aldrich (Merck). Materials used for prototype OFET devices were purchased from Sigma-Aldrich (Merck) and Ossila. Furthermore, 2,5-Diamino-thiophene-3,4-dicarboxylic acid diethyl ester (DAT) was synthesized according to publication [31].

### 3.2. Synthesis of the Thiophenoazomethines

Dialdehydes (2 mmol of: isophthalaldehyde, 4,4′-biphenyldicarboxaldehyde,4,4′-diformyltriphenylamine, 2,2′-bitiophene-5,5′-dicarboxaldehyde,thieno[3,2-b]thiophene-2,5-dicarboxaldehyde) were dissolved in 50 mLof ethanol in around bottom flask and heated to 78 °C. After 15 min2,5-diamino-thiophene-3,4-dicarboxylic acid diethyl ester (8 mmol; 2066 g) was added with four drops of trifluoroacetic acid (TFA). The reaction was carried out for 24 h, and after that time the product was filtered, dissolved in chloroform (20 cm^3^) with activated charcoal and filtered again to 10 cm^3^ of chloroform. The main product was received from the evaporated chloroform solution using a rotary evaporator.

***Bis-2-(1,3-iminophenylene)-5-amino*** ***-thiophene-3,4-dicarboxylic acid diethyl ester(AzDT-1)***

Yellow solid. Yield = 75%. ***^1^H NMR*** (δ, 600 MHz, DMSO-d_6_, ppm): 8.13 (s, 2H, H^–C=N−^), 8.11 (s, 1H), 7.95 (s, 4H, –NH_2_), 7.83 (d, *J = 7.8 Hz*, 2H), 7.56 (t, *J = 7.7 Hz*, 1H), 4.32 (q, *J = 7.1 Hz*, 4H), 4.16 (q, *J = 7.1 Hz*, 4H), 1.32 (t, *J = 7.1 Hz*, 6H),1.22 (t, *J = 7.1 Hz*, 6H). ***^13^C NMR*** (δ, 151 MHz, DMSO-d_6_, ppm): 165.1, 163.7, 161.7, 152.1, 136.7, 132.1, 131.0, 130.4, 129.8, 127.5, 100.6, 61.30, 60.0, 14.6, 14.5. ***FT-IR*** (KBr, *v*, cm^−1^): 3423, 3315 (NH_2_stretch), 3155 (C-H aromatic), 2976 (C-H aliphatic), 1741, 1706 (C=O), 1671, 1649 (CH=N stretch),1584 (C-N stretch). ***El.Anal. Calcd*** for C_28_H_30_N_4_O_8_S_2_ (614.69 g/mol): C (54.71%), H (4.92%), N (9.11%), ***Found:*** C (54.26%), H (4.36%), N (8.58%). ***HRMS*** (*m/z*) [M + Na]^+^calcd for C_28_H_30_N_4_NaO_8_S_2_: 637.1391. ρ = 1.39 g/cm^3^.

***Bis-2-(4,4′-diiminophenylene)-5-amino*** ***-thiophene-3,4-dicarboxylic acid diethyl ester (AzDT-2)***

Orange solid. Yield = 85%. ***^1^H NMR*** (δ, 600 MHz, DMSO-d_6_, ppm): 8.15 (s, 2H, H^–C=N−^), 7.93 (s, 4H, –NH_2_), 7.87 (s, 8H), 4.30 (q, *J = 7.1 Hz*, 4H),4.16 (q, *J = 7.1 Hz*, 4H), 1.34 (t, *J = 7.1 Hz*, 6H),1.22 (t, *J = 7.1 Hz*, 6H). ***^13^C NMR*** (δ, 151 MHz, DMSO-d_6_, ppm): 165.3, 163.8, 161.7, 152.0, 141.5, 135.8, 132.5, 130.7, 129.0, 127.5, 100.6, 61.2, 59.9, 14.6,14.5. ***FT-IR*** (KBr, *v*, cm^−1^): 3423, 3308 (NH_2_stretch), 3161 (C-H aromatic), 2988 (C-H aliphatic), 1715 (C=O), 1663 (CH=N stretch), 1593 (C-N stretch). ***El.Anal. Calcd*** for C_34_H_34_N_4_O_8_S_2_ (690.79 g/mol): C (59.12%), H (4.96%), N (8.11%), ***Found:*** C (58.88%), H (4.84%), N (7.61%). ***HRMS*** (*m/z*) [M + Na]^+^calcd for C_34_H_34_N_4_NaO_8_S_2_: 713.1703.ρ = 1.41 g/cm^3^.

***Bis-2-(4,4′-diiminotriphenylamine)-5-amino-thiophene-3,4-dicarboxylic acid diethyl ester (AzDT-3)***

Gold solid. Yield = 86%. ***^1^H NMR*** (δ, 600 MHz, DMSO-d_6_, ppm): 8.02 (s, 2H,H^–C=N−^), 7.87 (s, 4H, –NH_2_),7.69 (d, *J* *= 8.6 Hz*, 4H),7.41 (t, *J* *= 7.8 Hz*, 2H), 7.20 (t, *J* *= 7.4 Hz*, 1H), 7.14 (d, *J* *= 7.7 Hz*, 2H), 7.06 (d, *J* *= 8.6 Hz*, 4H),4.25 (q, *J = 7.1 Hz*, 4H), 4.14 (q, *J = 7.1 Hz*, 4H), 1.28 (t, *J = 7.1 Hz*, 6H),1.20 (t, *J = 7.1Hz*, 6H). ***^13^C NMR*** (δ, 151 MHz, DMSO-d_6_, ppm):165.3, 163.8, 161.2, 151.9, 149.1, 146.2, 132.9, 130.8, 130.4, 129.8, 129.5, 126.4, 125.5, 123.3, 100.5, 61.1, 59.9, 14.6, 14.5. ***FT-IR*** (KBr, *v*, cm^−1^): 3423, 3315 (NH_2_stretch), 3155 (C-H aromatic), 2976 (C-H aliphatic), 1741, 1706 (C=O), 1671, 1649 (CH=N stretch), 1584 (C-N stretch). ***El.Anal. Calcd*** forC_40_H_39_N_5_O_8_S_2_ (781.89 g/mol): C (61.44%), H (5.03%), N (8.96%), ***Found:*** C (60.81%), H (5.03%), N (8.52%). ***HRMS*** (*m/z*) [M + H]^+^calcd for C_40_H_40_N_5_O_8_S_2_: 782.2306. ρ = 1.32 g/cm^3^.

***Bis-2-(2,5-diimino-thieno[3,2-b]thiophene)-5-amino*** ***-thiophene-3,4-dicarboxylic acid diethyl ester (AzDT-4)***

Red solid. Yield = 81%. ***^1^H NMR*** (δ, 600 MHz, DMSO-d_6_, ppm): 8.29 (s, 2H, H^–C=N−^), 7.97 (s, 4H, –NH_2_), 7.86 (s, 2H), 4.29 (q, *J = 7.0 Hz*, 4H), 4.15 (q, *J = 7.1 Hz*, 4H), 1.34 (t, *J = 7.1 Hz*, 6H),1.22 (t, *J = 7.1 Hz*, 6H). ***^13^C NMR*** (δ, 151 MHz, DMSO-d_6_, ppm): 165.1, 163.6, 161.2, 147.2, 146.3, 141.9, 132.0, 130.7, 125.4, 100.8, 61.3, 60.1, 14.7, 14.5. ***FT-IR*** (KBr, *v*, cm^−1^): 3462, 3321 (NH_2_stretch), 2976 (C–H aliphatic), 1738, 1720 (C=O), 1692 (CH=N stretch), 1595 (C-Nstretch). ***El. Anal. Calcd*** for C_28_H_28_N_4_O_8_S_s_ (676.80 g/mol): C (49.69%), H (4.17%), N (8.28%), ***Found:*** C (48.97%), H (3.56%), N (7.68%). ***HRMS*** (*m/z*) [M + H]^+^calcd for C_28_H_29_N_4_O_8_S_2_: 677.0886.ρ = 1.45 g/cm^3^.

***Bis-2-(5,5′-diimino-2,2′-bitiophene)-5-amino*** ***-thiophene-3,4-dicarboxylic acid diethyl ester (AzDT-5)***

Red solid; Yield = 65%. ***^1^H NMR*** (δ, 600 MHz, DMSO-d_6_, ppm): 8.23 (s, 2H, H^–C=N−^), 7.95 (s, 4H, –NH_2_), 7.55 (d, *J = 3.9 Hz*, 2H), 7.49 (d, *J = 3.9 Hz*, 2H), 4.29 (q, *J = 7.0 Hz*, 4H), 4.15 (q, *J = 7.1 Hz*, 4H), 1.34 (t, *J = 7.1 Hz*, 6H),1.21 (t, *J = 7.1 Hz*, 6H). ***^13^C NMR*** (δ, 151 MHz, DMSO-d_6_, ppm): 165.1, 163.7, 161.5, 145.8, 142.5, 139.9, 133.8, 132.1, 130.3, 126.7, 100.7, 61.2, 60.0, 14.6, 14.5. ***FT-IR*** (KBr, *v*, cm^−1^): 3456, 3295 (NH_2_stretch), 2994 (C-H aliphatic), 1733 (C=O), 1669 (CH=N stretch), 1574 (C-N stretch). ***El. Anal. Calcd*** for C_30_H_30_N_4_O_8_S_4_ (702.84 g/mol): C (51.27%), H (4.30%), N (7.97%), ***Found:*** C (50.37%), H (3.93%), N (7.63%). ***HRMS*** (*m/z*) [M+H]^+^calcd for C_30_H_31_N_4_O_8_S_2_: 703.1039.ρ = 1.44 g/cm^3^.

## 4. Conclusions

The five azomethines with two free amino groups, able to form stable amorphous states, were synthesized in eco-friendly conditions. The impact of the imine core—phenyl, biphenyl, triphenylamine, thieno[3,2-b]thiophene and bithiophene—on thermal, optoelectronic and electrochemical properties was demonstrated. It was found that:

- the compounds both showed high T_m_ (above 200 °C) and T_g_ (above 100 °C). The presence of TPA and a biphenyl structure increase the T_m_ to ~300 °C and T_g_ to 194 °C (AzDT-3) and 163 °C (AzDT-2),
- the HOMO of the imines was in the similar range of −5.58–−5.15 eV, whereas in the LUMO value, due to the fact that it is mainly localized on the central molecule part, more pronounced differences were observed and molecules with TPA and phenyl unit exhibited the lowest LUMO energy level at −2.19 eV and −2.28 eV, respectively. The introduction of a phenyl and a biphenyl structure slightly increases the E_g_ from 1.5 eV to 1.6 eV,
- the presence of a thieno[3,2-b]thiophene and a bithiophenebathochromically shifted the absorption range and together with TPA have a beneficial effect on PL efficiency. The weak emission in the solutions and its lack in the thin films is related to the presence of a heavy atom (sulfur) and internal conversion,
- imines smoothed the perovskite layer, which improves the HTM—electrode interfacial contact,
- low value of FF and *J_sc_* of the fabricated hybrid solar cells based on the synthesized azomethines have resulted in poor power conversion efficiency, not excited 3.65%.

The presented molecules are potential monomers because of two free amine groups, and the most promising ones as examples with a TPA unit will be applied for polycondensation for the preparation of the conjugated polymers in further investigations.

## Figures and Tables

**Table 1:** Thermal properties of the investigated compounds.

Code	DSC		
	I Heating	II Heating	
	Scan	Scan	
	T_m_	T_g_	T_m_
	[°C]	[°C]	[°C]
AzDT-1	118, 242	101	nd
AzDT-2	118, 300	163	300
AzDT-3	262	194	nd
AzDT-4	117, 297	157	nd
AzDT-5	118, 260	137	nd

T_m_—melting temperature, T_g_—glass transition temperature, nd—not detected.

**Table 2:** The redox properties of the thiophenoazomethines.

Code	Method	E_red_^1^	E_red(onset)_^1^	E_ox_^1^	E_ox(onset)_^1^	EA	LUMO^c^	IP	HOMO ^c^	E_g_
		(V)	(V)	(V)	(V)	(eV)	(eV)	(eV)	(eV)	(eV)
AzDT-1	DPV	−1.81	−1.33	0.42	0.28	−3.77	−2.28	−5.38	−5.52	1.61
	CV	−1.84 ^a^	−1.29	0.52 ^a^	0.36	−3.81		−5.46		1.65
AzDT-2	DPV	−1.82	−1.39	0.63	0.28	3.71	−2.48	−5.38	−5.41	1.67
	CV	−1.73 ^a^	−1.3	0.38 ^a^	0.34	−3.8		−5.44		1.64
AzDT-3	DPV	−1.78	−1.45	0.21	0.07	−3.65	−2.19	−5.17	−5.24	1.52
	CV	−1.78 ^a^	−1.50	0.27 ^a^	0.14	−3.6		−5.24		1.64
AzDT-4	DPV	−1.58	−1.27	0.29	0.18	−3.83	−2.81	−5.28	−5.28	1.45
	CV	−1.56 ^a^	−1.24	0.34 ^a^	0.27	−3.86		−5.37		1.51
AzDT-5	DPV	−1.65	−1.35	0.37	0.15	−3.75	−2.74	−5.25	−5.15	1.5
	CV	−1.63 ^a^	−1.28	0.42 ^b^	0.25	−3.82		−5.35		1.53

IP= −5.1 − E_ox(onset)_·|e^−^|, EA= −5.1 − E_red(onset)_·|e^−^|, E_g_ = E_ox(onset)_ − E_red(onset)_. Measurements in CH_2_Cl_2_ with concentration 10^−3^ mol/dm^3^ and electrolyte 0.1 mol/dm^3^ Bu_4_NPF_6_. Pt as the working electrode. ^a^ Irreversible process. ^b^ Quasi-reversible process. v = 0.1 V/s for CV and v = 0.01 V/s for DPV. ^c^ Data from the DFT calculations. ^1^ The first reduction and oxidation processes.

**Table 3:** UV-Vis and PL data of AzDTs.

Code	Medium	UV-Vis	PL		Φ (%)
		*λ_max_*	*λ_em_*	Stokes Shifts	
		(nm) (ε·10^4^) ^a^	(nm)	(cm^−1^) ^b^	
AzDT-1	CHCl_3_^c^	401 (4.05)	450	2715	0.2
	CH_2_Cl_2_	404 (3.88)	453	2677	0.1
	C_6_H_5_Cl	401 (2.68)	-	-	-
	C_2_H_3_N	211 (4.41), 242 (3.32), 410 (4.51)	468	3023	0.1
	Film ^d^	407	-	-	-
AzDT-2	CHCl_3_	304 (2.29), 428 (6.28)	494	3122	0.1
	CH_2_Cl_2_	308 (1.74), 431 (4.76)	496	3041	0.2
	C_6_H_5_Cl	310 (2.33), 433 (5.76)	494	2852	0.2
	C_2_H_3_N	295 (2.32), 431 (5.63)	530	4334	0.2
	Film ^d^	445	-	-	-
AzDT-3	CHCl_3_	306 (2.56), 404 ^sh^, 445 (7.52)	500	2472	2.4
	CH_2_Cl_2_	309 (2.59), 404 ^sh^, 445 (5.54)	506	2709	2.3
	C_6_H_5_Cl	407 ^sh^, 446 (6.96)	501	2461	2.0
	C_2_H_3_N	242 (3.69), 310 (2.00), 401 ^sh^, 442 (6.92)	507	2901	0.5
	Film ^d^	454	-	-	-
	Film ^e^	449	-	-	-
AzDT-4	CHCl_3_	325 (1.62), 459 ^sh^, 492 (5.58), 527 (4.88)	564	1245	1.8
	CH_2_Cl_2_	266 (1.59), 322 (1.59), 459 ^sh^, 489 (6.83), 525 (5.94)	564	1317	0.9
	C_6_H_5_Cl	325 (1.55), 461 ^sh^, 494 (2.57), 533 (2.34), 601 ^sh^	560,591	905	1.2
	C_2_H_3_N	220 (5.46), 325 (1.77), 487 (6.42), 521 (5.69)	595	2387	1.0
	Film ^d^	495	-	-	-
AzDT-5	CHCl_3_	272 (2.62), 404 ^sh^, 500 (6.58)	582	2818	2.0
	CH_2_Cl_2_	272 (2.38), 311 ^sh^, 401 ^sh^, 501 (6.52)	585	2866	1.2
	C_6_H_5_Cl	406 ^sh^, 474 ^sh^, 507 (1.57), 542 ^sh^	578,616	2423	1.0
	C_2_H_3_N	219 (4.64), 272 (2.11), 404 ^sh^, 501 (5.35)	612	3620	1.3
	Film ^d^	489	-	-	-

^a^ ε—Absorption coefficient (dm^3^·mol^−1^·cm^−1^). ^b^ Stokes shifts calculated according to the equation Δν = (1/λ_abs_ − 1/λ_em_)·10^7^ (cm^−1^). ^c^ Concentration of the solutions= 10^−5^ mol/dm^3^. ^d^ Film prepared from chloroform solution. ^e^ Film prepared from chlorobenzene solution. ^sh^—shoulder. Underline data indicates the excitation wavelength (λ_ex_).

**Table 4:** Photovoltaic properties of the best fabricated hybrid perovskite solar cells: FTO/b-TiO_2_/m-TiO_2_/perovskite/Au, and TiO_2_/perovskite/AzDTs:V_Li-TFSI_/Au.

Code	V_Li-TFSI_ (μL)	*J_sc_*	*V_oc_*	FF	PCE
		(mA/cm^2^)	(mV)	(-)	(%)
reference	-	9.24	156.10	0.25	0.41
spiro-OMeTAD	17.50	15.35	739.80	0.42	5.05
AzDT-1	8.75	3.88	603.90	0.41	1.10
	17.50	3.68	425.70	0.33	0.59
	35.00	6.72	530.30	0.34	1.37
AzDT-2	8.75	7.60	760.80	0.34	2.24
	17.50	6.52	759.50	0.42	2.38
	35.00	3.92	301.10	0.31	0.42
AzDT-3	8.75	13.50	673.50	0.35	3.64
	17.50	7.80	680.50	0.35	2.11
	35.00	2.80	513.90	0.35	0.57
AzDT-4	8.75	3.92	708.10	0.46	1.46
	17.50	3.68	758.60	0.59	1.88
	35.00	4.80	778.90	0.50	2.14
AzDT-5	8.75	5.00	798.00	0.45	2.04
	17.50	3.28	753.40	0.41	1.16
	35.00	1.72	640.30	0.33	0.42

## Data Availability

The data are available in this publication and Supplementary Materials.

## Supplementary Materials

The following supporting information can be downloaded at: https://www.mdpi.com/article/10.3390/ijms23158160/s1. References [44,64,65,66,67,68,69,70] are cited in the Supplementary Materials.
