# Supplementary material for: Synthesis and Characterization of New Conjugated Azomethines End-Capped with Amino-thiophene-3,4-dicarboxylic Acid Diethyl Ester
Source: Int J Mol Sci. 2022 Jul 24;23(15):8160. doi: 10.3390/ijms23158160 (PMC9330727; doi:10.3390/ijms23158160)
Supplement: Supplementary file 1 [file ijms-23-08160-s001.zip › ijms-1817930-supplementary.pdf]

# Supplementary Materials (ESI)

## Synthesis and characterization of new conjugated azomethines end capped with amino-thiophene-3,4-dicarboxylic acid diethyl ester

Agnieszka Katarzyna Pająk[1,2], Sonia Kotowicz[1,*], Paweł Gnida[2], Jan Grzegorz Małecki[1], Agnieszka Ciemięga[3], Adam Łuczak[4], Jarosław Jung[4], Ewa Schab-Balcerzak[1,2,*]

[1]*Institute of Chemistry, University of Silesia, 9 Szkolna Str., 40-006 Katowice, Poland*
[2]*Centre of Polymer and Carbon Materials, Polish Academy of Sciences, 34 M. Curie-Sklodowska Str., 41-819 Zabrze, Poland*
[3]*Institute of Chemical Engineering, Polish Academy of Sciences, 5 Bałtycka Str., 44-100 Gliwice, Poland*
[4]*Departement of Molecular Physics, Faculty of Chemistry, Lodz University of Technology, 116 Żeromskiego Str., 90-924 Lodz, Poland*

**Table of contents**

1. **Solid state and hybrid perovskite solar cell preparations** 2
2. **Characterization Methods** 2
3. **DFT calculations** 3
4. **Dielectric constant calculations** 4
5. **OFET substrate preparations** 4
6. **Measurements of current-voltage characteristic of prototype OFET** 6
7. **Pictures of the investigated compounds under day light** 8
8. **$^{1}$H NMR and $^{13}$C NMR** 7
9. **DSC thermograms** 8
10. **Electrochemical data** 9
11. **Theoretical calculations** 10
12. **UV-Vis spectra** 16
13. **PL spectra** 17
14. **The root-mean-square parameter** 17
15. **SEM images** 18
16. **Solar cell data** 19
17. **Density and dielectric constant** 21
18. **OFET current-voltage characteristic** 21

## 1. Solid state and hybrid perovskite solar cell preparations

The films were prepared by spin-coating (1000 rpm, 60s;Laurell WS 650MZ-23NPPB) on the glass substrates from a homogeneous chloroform or chlorobenzene solutions (10 mg/mL).

The FTO glass slides (2 x 2 cm) were cleaned with surfactant Hellmanex III (Hellma Analytics), deionized water, and IPA (isopropanol, POCH) by ultrasonication. Following, a blocking layer b-$TiO_2$ and mesoporous m-$TiO_2$ layer were supplied on the cleaned FTOs as described in paper [1]. Perovskite layer was deposited by the two-step method.In the first step, the hot solution of $PbI_2$ in anhydrous DMF (N,N-dimethylformamide, 400 mg/mL) was spin-coated on the FTO with $TiO_2$ layers at 2000 r.p.m. for 30 seconds. Subsequently, the samples were dried at 70 °C for 3 minutes and 90 °C for 5 minutes. In the second step, the samples were dipped in a solution of MAI in IPA (9.09 mg/mL, using prewetting by IPA) and the samples were then dried at 90 °C for 30 minutes. The composition of imino-bis-thiophene derivatives' mixture was $5.9 \cdot 10^{-5}$mol of the synthesized compoundin 1 mLof chlorobenzene, 8.75 µL or 17.5 µL or 35 µL of lithium bis(trifluoromethanesulfonyl)imide (0.002 mol/mL Li-TFSI in acetonitrile) and 28.8 µL of 4-tert-butyl pyridine. The solution of thiophene compound was spin-coated on the FTO/b-$TiO_2$/m-$TiO_2$ (at 4.000 r.p.m. for 30 s). The thermal evaporation was used to obtain the gold electrode on the FTO/b-$TiO_2$/m-$TiO_2$/perovskite/HTM (~$10^{-6}$ mbar).

## 2. Characterization methods

Nuclear magnetic resonance spectra were recorded on a Bruker Avance II Ultrashield Plus 600 MHz (Germany) spectrometer in DMSO-$d_6$ as a solvent. The elementary analysis was measured using Vario EL III apparatus (Elementar, Germany). FTIR absorption spectrum were recorded on a Thermo Scientific Nicolet iS5 FT-IR Spectrometer in the range of 4000 - 400 $cm^{-1}$ as KBr pressed pellets (KBr before use was dried). Products were evaporated on IKA RV8 with VACOSTAR CONTROL. The synthesis were performed using IKA C-MAG HS7 and VWR VMS-C7 hot plates. Spectrometer ESI-Q-TOF, maXis impact Bruker Daltonics were used to registered HRMS spectras with HR-ESI/APCI-MS method. Thermal instigationswere performed using a DSC Q2000 TA Instruments with a heating/cooling rate of 20ºC·$min^{-1}$ under nitrogen and using aluminum sample pans in the range of 0 – 300 °Cand a Mettler Toledo TGA STARe system with a heating rate of 10°C·$min^{-1}$ in a constant stream of nitrogen (20 ml·$min^{-1}$) and a temperature range from 50°C to 600°C. The glass transition

temperature ($T_g$) was recorded in the second heating scan after first heating scan and cooling scan. Absorption spectra were performed using an Evolution 220 UV-Visible Spectrophotometer with1 cm quartz cell and Jasco V-550 Spectrophotometer for films. The Varian Carry Eclipse Spectrometer was used to recorded photoluminescence spectra in solutions and the Hitachi F-2500 Spectrometer forrecordedfilms photoluminescence spectra. Quantum yields ($\Phi_{PL}$) measurements were performed by using the integrating sphere AvantesAvaSphere-80 (Edinburgh Instruments) and absolute method.Electrochemical measurements were performed with Eco ChemieAutolab PGSTAT128n potentiostat in a one-compartment cell in DCM (dichloromethane)(Sigma-Aldrich (Merck) for HPLC, 99.8%). A platinumwire (diam. 2.0 mm) was served as a workingelectrodethe platinum coil and silver wire were used as auxiliary and reference electrode, respectively. As the supporting electrolyte salt the$Bu_4NPF_6$ (Aldrich, 99%) was used with the concentration 0.1mol/dm$^3$. Each experiment was performed in an air-conditioned room at 20°C±1°C and under argon purging. The measurements were recorded with moderate scan rate equal to 0.1 V/s for CV and 0.01 V/s for DPV. All potentials were referenced to the stable $Fc/Fc^+$ couple (and the IP of ferrocene couple was calculated to be equal to -5.1 eV) [2].The surface morphology of films and electrodes in nanoscale was characterized by atomic force microscopy (AFM) using TopoMetrix Explorer device, operating in contact mode, in air, in constant force regime. The piezoelectric scanner had a scan range of approximately 20 µm x 20 µm.The surfaces and cross-sectional SEM images were taken using a SEM microscope (Quanta/FEG 250/FEI Co.). The prepared devices were tested using the PET Photo Emission Tech Inc. Model SS 200AAA class solar simulator in STC (Standard Test Conditions) (1000 W/m$^2$, 25°C, AM1.5). The examined active area was 0.25 cm$^2$.The absolute density of the presented new compounds was measured using a helium pycnometer AccuPyc 1330 Micrometrics after 2h in the 100°C in an oven in the standard conditions.

## 3. DFT calculations

The theoretical calculations were performed with the use of the density functional theory (DFT) and were carried out using the Gaussian09 program [3] on B3LYP/6-311g++ level [4,5]. Molecular geometry of the singlet ground state of the compounds was optimized in the gas phase and the frequency calculation for each of the compounds shows only positive values which verify that the optimized molecular structure corresponds to energy minimum (experimental and calculated IR spectra are presented in the Fig. S5.).Solvent effect was taken into account using polarizable continuum model (PCM) [5] with dichloromethane and

chlorobenzene as solvents. Such calculations were carried out for analysis of the frontier molecular orbitals structure, energy levels and UV-Vis data. The optimized geometries of the compounds are depicted inthe Fig. S6. Density of states diagram were obtained with use of GaussSum program [6]. The TD-DFT (time dependent density functional theory) method [7] was employed to calculate the electronic absorption spectra of the compounds in chloroform and geometries in singlet/triplet excited states.

## 4. Dielectric constant calculations

Calculations were made use the B3lyp/6-311+G(d,p)level of theory augmented with GD3BJ dispersion correction model [8]. The continuum solvation model PCM was used to describe the solvent (trichlorobenzene) effect. Molecular volumes were obtained with use of tight option for better accuracy. The dielectric constant $\varepsilon_r$was obtained by Clausius–Mossotti equation, written as follows:

$$\frac{\varepsilon_r - 1}{\varepsilon_r + 2}\frac{M_N}{\rho} = \frac{N_A \alpha}{3\varepsilon_0}$$

where $\varepsilon_r$is the dielectric constant, $M_N$ is the molar mass of the dielectric medium, and $\rho$ is its density. $N_A$ is the Avogadro's constant, $\alpha$ is the polarizability, and $\varepsilon_0$ is the permittivity of free space. The dielectric constants are presented in the Table S6.

## 5. OFET substrate preparations

Transistors were preparate using Ossila pre-patterned ITO OFET substrates with ITO (d = 100 nm, 20Ω/square) with size 20 mm x 15 mm and channel dimensions 30 mm x 50 µm. The ITO substrate have been cleaned in hot sodium hydroxide (NaOH, BioXtra, >98%, 10%, sonicate) for 5 minutes, than have been dump rinses in hot water (DI) and sonicate in hot 2% Hellmanex III (purchase from Ossila) for 5 minutes. After that time the substrates have been dump rinses in hot water (DI) and cold water (DI) and sonicate in cold 2-propanol (anhydrous, 99.5%) for 5 minutes. To dry the substrates inert gas was used (argon). Organic material (10mg) act as active layer was dissolved in 1cm$^3$ 1,2,4-trichlorobenzene (TCB, for HPLC, >99%) and filtered with a 0.45 µm PTFE membrane filter. A 30 µl of prepared organic material was static dispense on ITO OSSILA substrate. On the active layer the poly(methyl methacrylate) (PMMA, Mw ≈ 120 000 by GPC, dissolved in butan-2-one at 100mg/cm$^3$)was dynamic dispensed with 1000 rpm for 30 seconds (d = 1 µm). PMMA was

scratch from gate and contact pads using precision tweezers and then baked at 105°C for 5 minutes. PMMA was used as an gate insulator andPH 1000 PEDOT:PSS was used as gate electrode. PEDOT:PSS, N-methyl-2-pyrrolidone (NMP, for HPLC, >99%), triton X-100 and 2-propanol were mixed together with proportions 42.5% by weight, 2.5% by weight, 1% by weight, 54% by weight, respectively. A 50μl PEDOT:PSS mixture was dispense on PMMA. The device was fabricated in the chamber blew with argon before use at room temperature and humidity below 15%.

## 6. Measurements of current-voltage characteristic of prototype OFET

The current-voltage characteristics of OFET were taken using a Keithley 2636B SMU instrument with Ossila test board at room temperature and humidity below 15% in the air atmosphere without the presence of light. The output characteristics of OFETs with active layers of P3HTand AzDT-3were measured in the range of 0 - -40V (see Fig. S13). The charge carrier mobilities were determined on the basis of transfer current-voltage characteristics at the saturation regime of OFETs using formula:

$$\mu_{FET} = \frac{2\alpha^2 L}{W C_p}$$

where $\alpha$ is the slope of the linear part of the graph $I_{DS}^{1/2}=f(U_{GS})$, L is the channel length, W is the channel width and $C_p$ is the capacitance per area unit of the gate dielectric. For template OFET with P3HT active layer determined mobility of charge carriers was $\mu_{FE\ T}= 1.65 \cdot 10^{-3}$ $cm^2V^{-1}s^{-1}$. The measurements were taken with $V_{gs} < 0$ and $V_{ds} < 0$.

[1 (44)] Pająk A., Gnida P., Kotowicz S., et al., Energy Fuels, 2020, 34, 10160-10169.
[2 (64)] BujakP., Kulszewicz-BajerI., Zagorska M., et.al., Chem. Soc. Rev. 2013, 42, 8895–8999.
[3 (65)] Gaussian 09, RevisionA.02, Frisch M. J., Trucks G. W., Schlegel H. B., Scuseria G. E., Robb M. A., Cheeseman J. R., Scalmani G., Barone V., Petersson G. A., Nakatsuji H., Li X., Caricato M., Marenich A., Bloino J., Janesko B. G., Gomperts R., Mennucci B., Hratchian H. P., Ortiz J. V., Izmaylov A. F., Sonnenberg J. L., Williams-Young D., Ding F., Lipparini F., Egidi F., Goings J., Peng B., Petrone A., Henderson T., Ranasinghe D., Zakrzewski V. G., Gao J., Rega N., Zheng G., Liang W., Hada M., Ehara M., Toyota K., Fukuda R., Hasegawa J., Ishida M., Nakajima T., Honda Y., Kitao O., Nakai H., Vreven T., Throssell K., Montgomery J. A. Jr., Peralta J. E., Ogliaro F., Bearpark M., Heyd J. J., Brothers E., Kudin K. N., Staroverov V. N., Keith T., Kobayashi R., Normand J., Raghavachari K., Rendell A., Burant J. C., Iyengar S. S., Tomasi J., Cossi M., Millam J. M., Klene M., Adamo C., Cammi R., Ochterski J. W., Martin R. L., Morokuma K., Farkas O., Foresman J. B., and D. J. Fox, Gaussian, Inc., Wallingford CT, 2016.
[4 (66)] Becke A. D., J.Chem.Phys., 1993,98, 5648-5652.
[5 (67)] Lee C., Yang W., Parr R.G., Phys. Rev. B., 1988,37, 785-789.

[6 (68)] O'BoyleN.M.,TenderholtA.L.,LangnerK.M., J. Comp. Chem. 2008, 29, 839-845.
[7 (69)] CasidaM.E., in: J.M. Seminario (Ed.), Recent Developments and Applications of Modern Density Functional Theory, Theoretical and Computational Chemistry, vol. 4, Elsevier, Amsterdam, 1996, p. 391.
[8 (70)] GrimmeS., EhrlichS.,GoerigL.,J. Comp. Chem.,2011, 32, 1456-1465.

## 7. Pictures of the investigated compounds under day light

**a)**

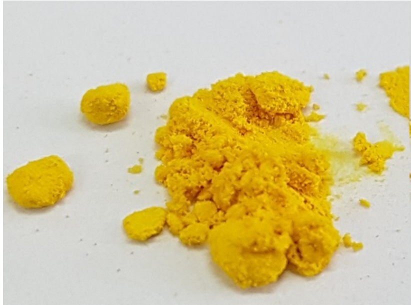

**b)**

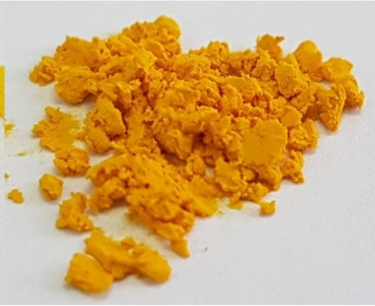

**c)**

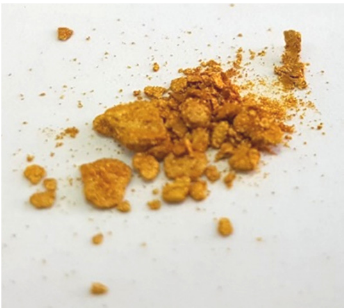

**d)**

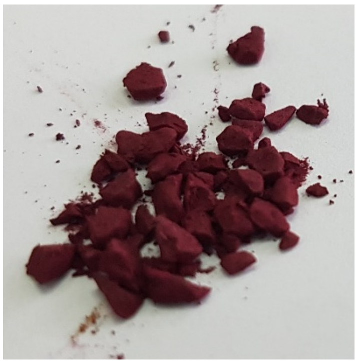

**e)**

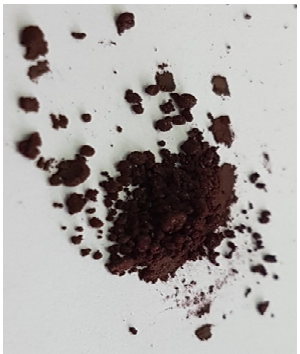

Fig. S1.Pictures of the investigated compounds under day light, a) AzDT-1, b) AzDT-2, c) AzDT-3, d) AzDT-4 and e) AzDT-5.

# 8. $^{1}$H NMR spectra

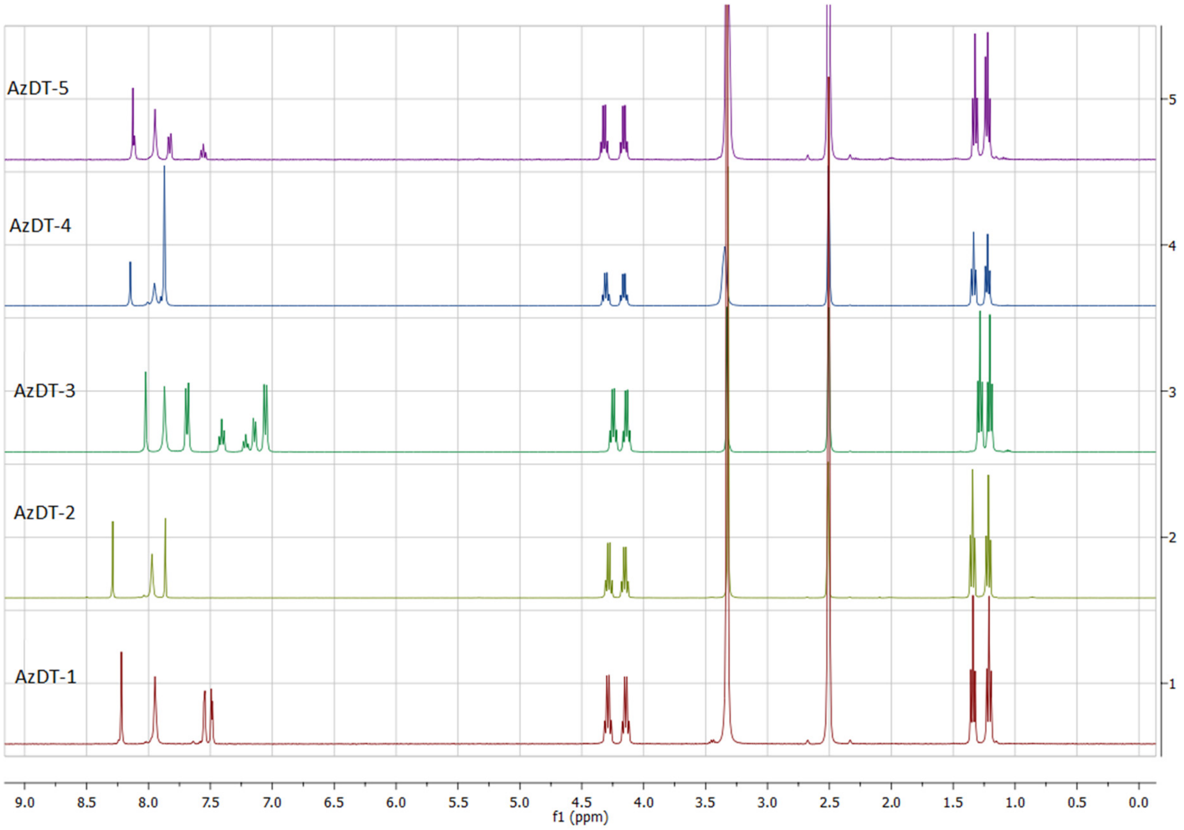


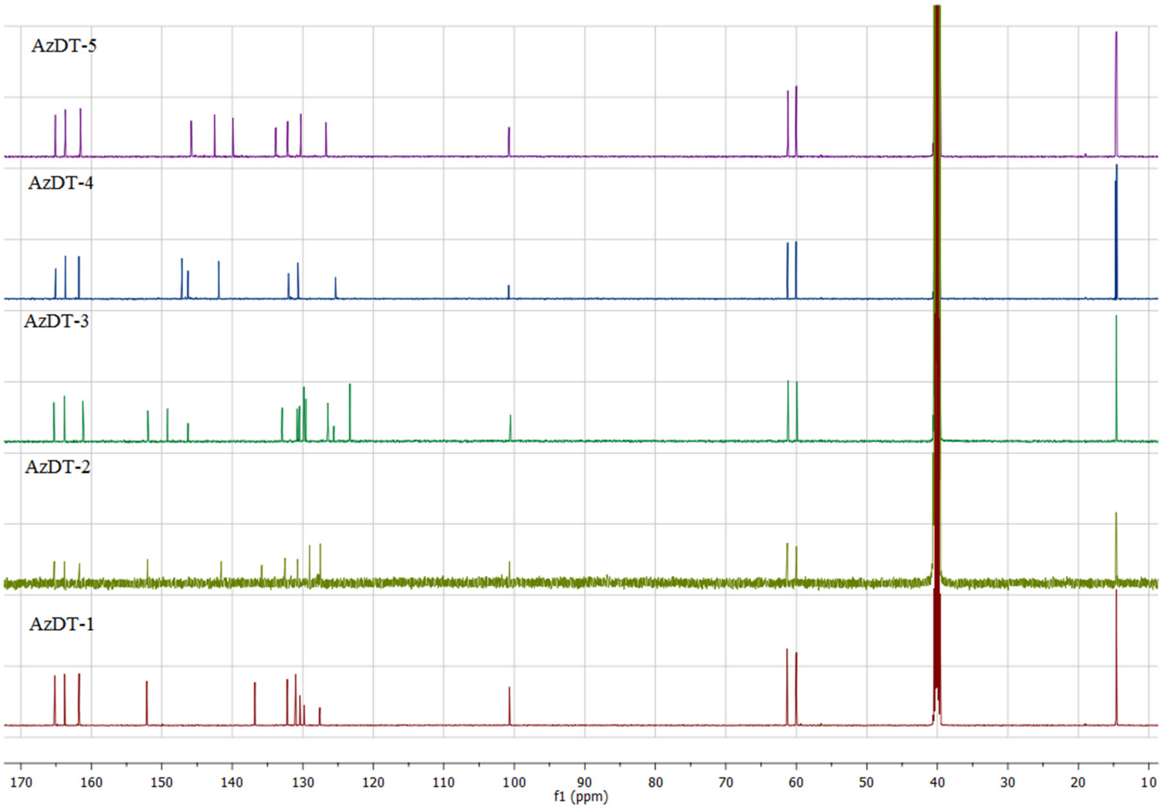


Fig. S2.$^{1}$HNMR (600 MHz) and $^{13}$C NMR (151 MHz)of the investigated compounds (DMSO-d6).

# 9. DSC thermograms

a)

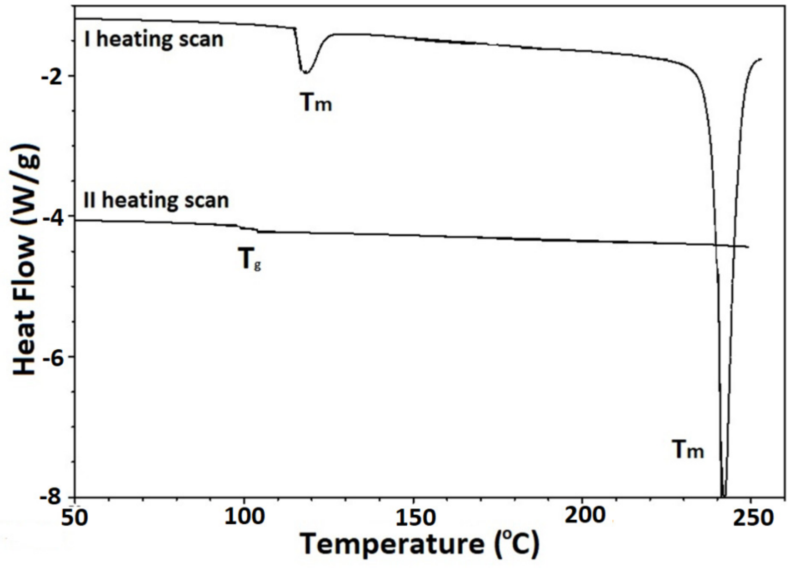


b)

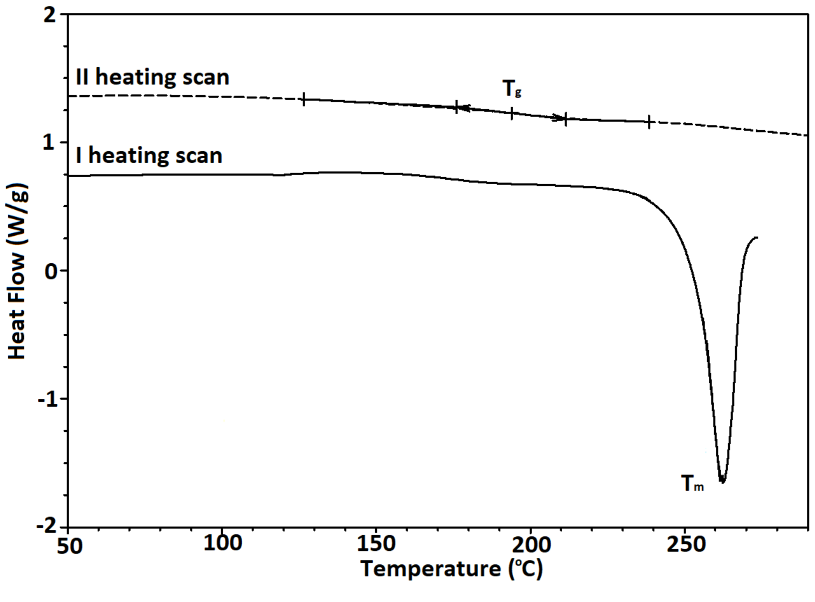


Fig. S3. DSC thermograms of a) AzDT-1 and b) AzDT-3registered in the first and second heating scan.

## 10. Electrochemical data

Table S1. The redox properties of the imino-bis-thiophene derivatives with the optical band gap.

| Code | Method | $E_{red}^{1}$ [V] | $E_{red}^{2}$ [V] | $E_{red}^{3}$ [V] | $E_{ox}^{1}$ [V] | $E_{ox}^{2}$ [eV] | $E_{ox}^{3}$ [eV] | $E_{g}^{opt}$ [eV] |
|---|---|---|---|---|---|---|---|---|
| AzDT-1 | DPV | -1.81 | -2.16 | - | 0.42 | - | - | 2.78 |
| | CV | -1.84[a] | -2.15[b] | - | 0.52[a] | - | - | |
| AzDT-2 | DPV | -1.82 | -2.11 | -2.53 | 0.63 | - | - | 2.55 |
| | CV | -1.73[a] | -1.88[a] | -2.05[a] | 0.38[a] | 0.47[a] | - | |
| AzDT-3 | DPV | -1.78 | -2.23 | - | 0.21 | 0.38 | 0.51 | 2.48 |
| | CV | -1.78[a] | -2.23[a] | - | 0.27[a] | 0.45[a] | 0.63[a] | |
| AzDT-4 | DPV | -1.58 | -1.82 | -2.33 | 0.29 | 0.51 | - | 2.21 |
| | CV | -1.56[a] | -1.87[a] | - | 0.34[a] | 0.57[a] | - | |
| AzDT-5 | DPV | -1.65 | -1.89 | -2.37 | 0.37 | - | - | 2.16 |
| | CV | -1.63[a] | -1.91[a] | - | 0.42[b] | - | - | |

[a]Irreversible process. [b]Quasi-reversible process. V=0.1V/s for CV and v=0.01V/s for DPV.$CH_2Cl_2$ with concentration $10^{-3}$ mol/$dm^3$ and electrolyte 0.1 mol/$dm^3$ $Bu_4NPF_6$. Pt as the working electrode. Optical band gap from $CH_2Cl_2$ solution.

a) b)

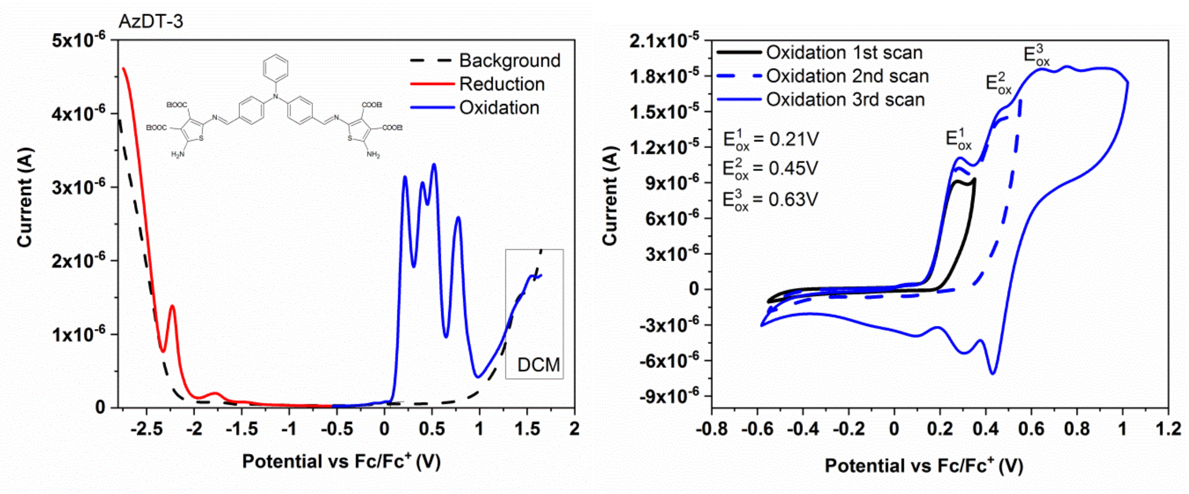


Fig. S4. a) The DPV voltammograms of AzDT-3 (oxidation and reduction) and b) the oxidation process of AzDT-3from CV measurements.

# 11. Theoretical calculations

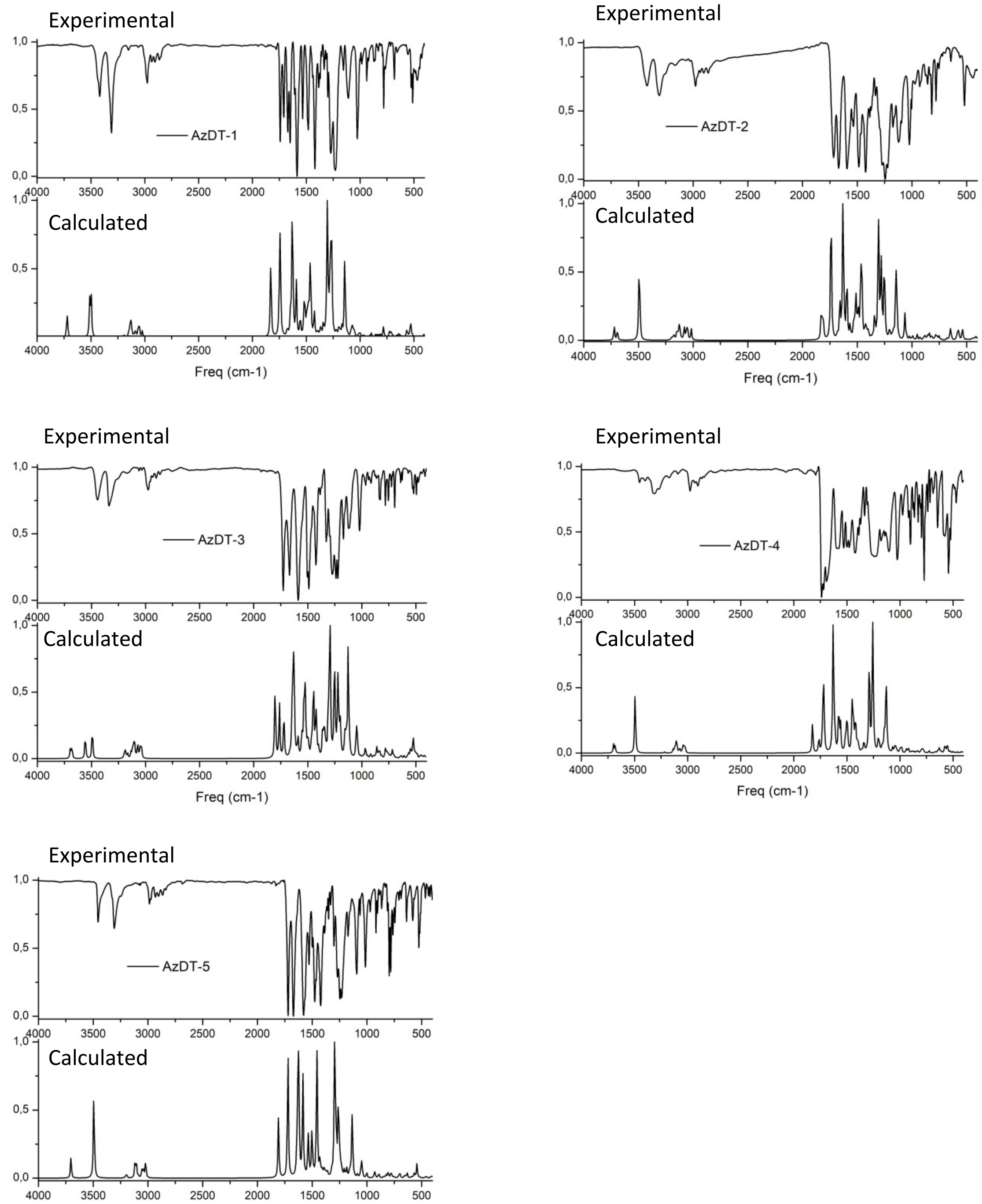


Fig. S5. Normalized experimental and calculated IR spectra.

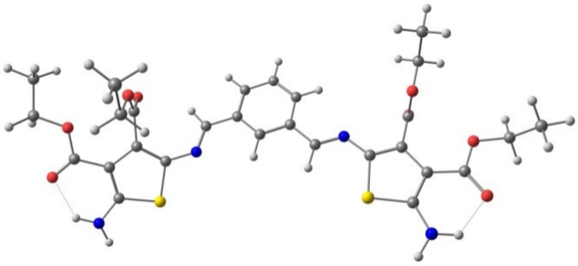

AzDT-1

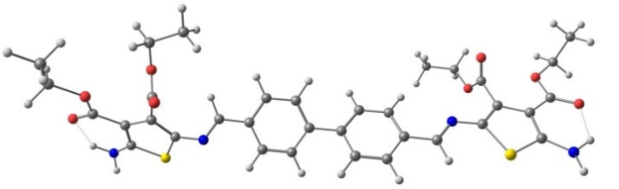

AzDT-2

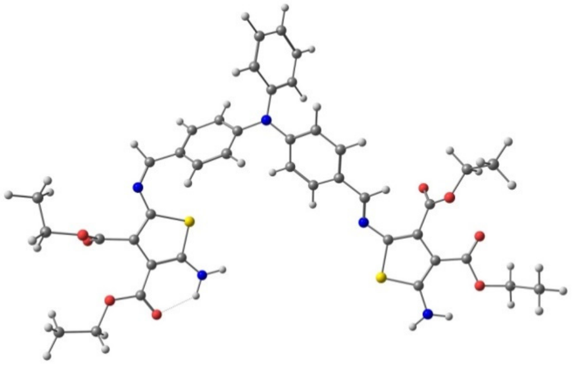

AzDT-3

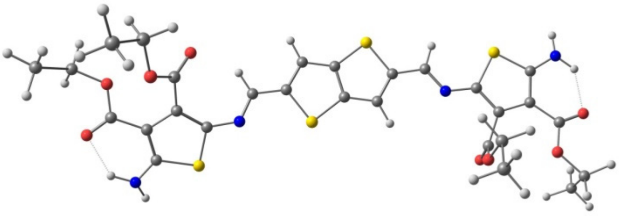

AzDT-4

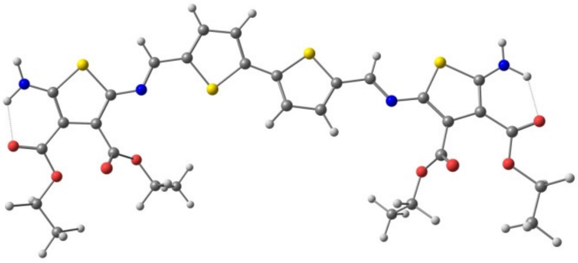

AzDT-5

Fig. S6. Optimized geometries of the compounds.

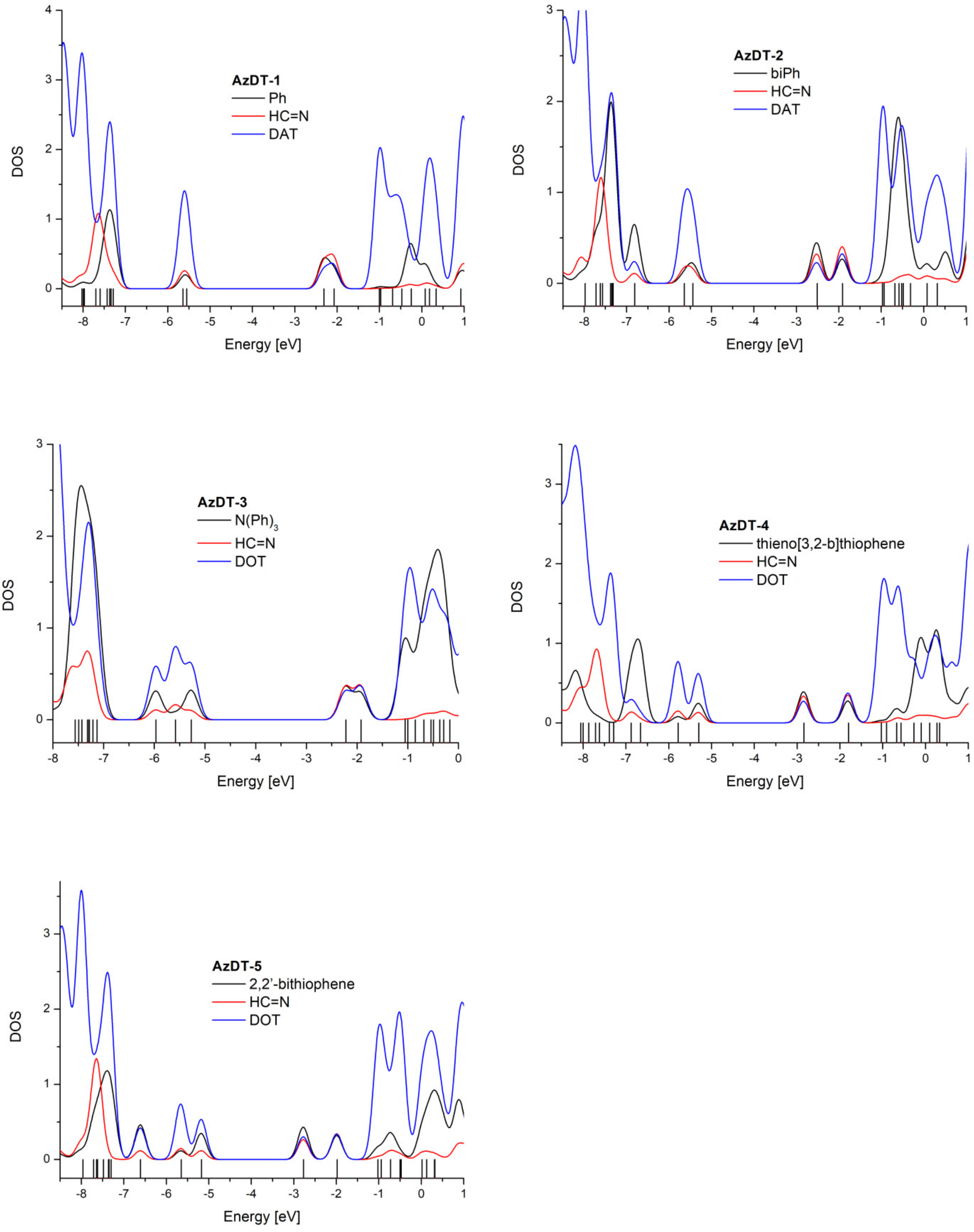


Fig. S7. Density-of-states diagrams.

| | HOMO | LUMO |
|---|---|---|
| AzDT-1 | 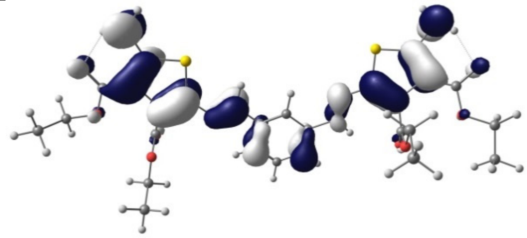 | 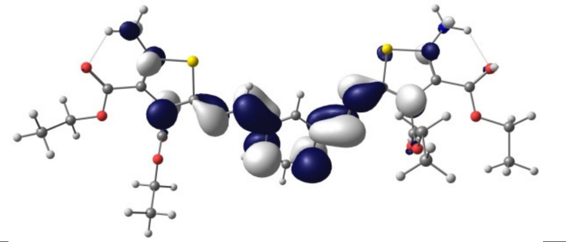 |
| AzDT-2 | 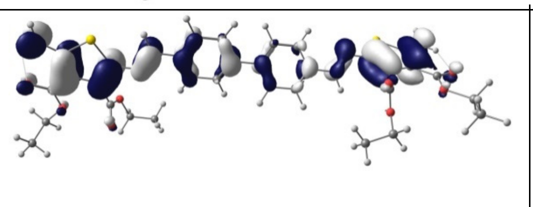 | 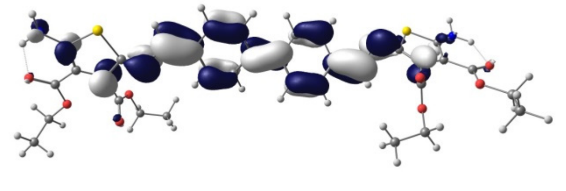 |
| AzDT-3 | 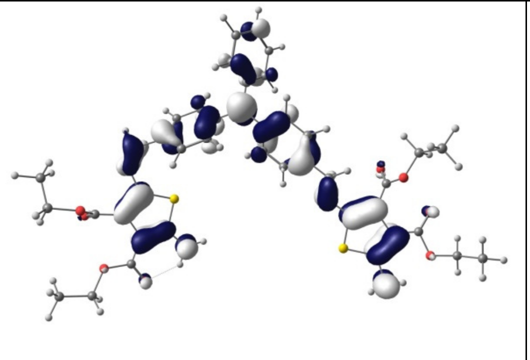 | 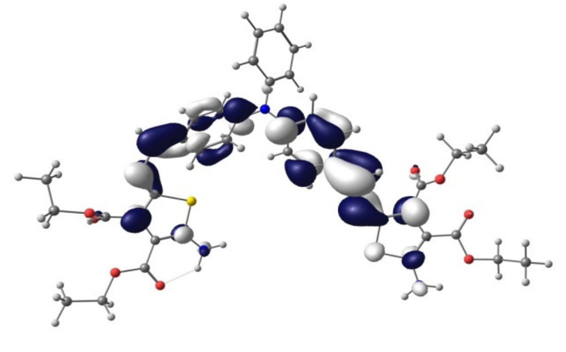 |
| AzDT-4 | 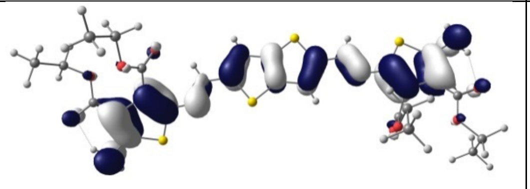 | 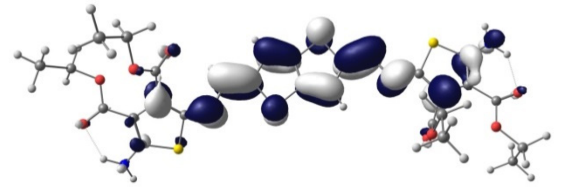 |
| AzDT-5 | 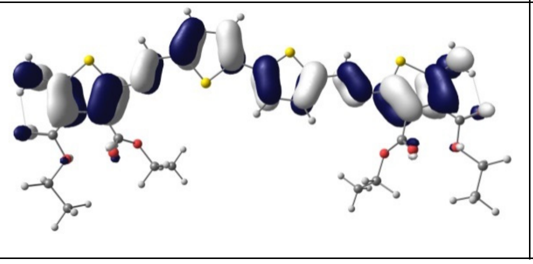 | 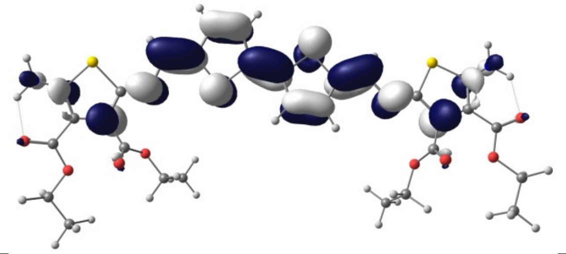 |

Fig. S8. Contours of HOMO and LUMO.

Table S2. Ground state composition of selected molecular orbitals.

| **AzDT-1** | eV | Ph | HC=N | DAT | **AzDT-2** | eV | bPh* | HC=N | DAT |
|---|---|---|---|---|---|---|---|---|---|
| L+5 | -0.47 | 1 | 2 | 97 | L+5 | -0.58 | 22 | 4 | 75 |
| L+4 | -0.69 | 2 | 1 | 97 | L+4 | -0.68 | 91 | 0 | 9 |
| L+3 | -0.97 | 3 | 0 | 97 | L+3 | -0.93 | 1 | 0 | 98 |
| L+2 | -1.00 | 0 | 0 | 100 | L+2 | -0.97 | 4 | 1 | 96 |
| L+1 | -2.07 | 26 | 42 | 32 | L+1 | -1.91 | 27 | 40 | 33 |
| LUMO | -2.31 | 40 | 35 | 25 | LUMO | -2.51 | 45 | 32 | 23 |
| HOMO | -5.55 | 14 | 13 | 72 | HOMO | -5.44 | 20 | 13 | 67 |
| H-1 | -5.64 | 7 | 14 | 78 | H-1 | -5.64 | 9 | 14 | 77 |
| H-2 | -7.29 | 64 | 16 | 20 | H-2 | -6.81 | 65 | 11 | 24 |
| H-3 | -7.34 | 0 | 1 | 99 | H-3 | -7.32 | 3 | 1 | 97 |
| H-4 | -7.37 | 0 | 1 | 99 | H-4 | -7.34 | 91 | 0 | 9 |
| H-5 | -7.43 | 67 | 9 | 24 | H-5 | -7.35 | 7 | 1 | 92 |

| **AzDT-3** | eV | $NPh_3$ | HC=N | DAT | **AzDT-4** | eV | tt* | HC=N | DAT |
|---|---|---|---|---|---|---|---|---|---|
| L+5 | -0.68 | 80 | 4 | 15 | L+5 | -0.57 | 1 | 2 | 96 |
| L+4 | -0.85 | 17 | 1 | 82 | L+4 | -0.67 | 17 | 4 | 79 |
| L+3 | -1.00 | 2 | 0 | 98 | L+3 | -0.91 | 0 | 0 | 100 |
| L+2 | -1.05 | 81 | 0 | 19 | L+2 | -1.03 | 3 | 1 | 96 |
| L+1 | -1.92 | 29 | 36 | 36 | L+1 | -1.80 | 28 | 35 | 37 |
| LUMO | -2.22 | 35 | 35 | 30 | LUMO | -2.84 | 39 | 34 | 27 |
| HOMO | -5.27 | 32 | 10 | 58 | HOMO | -5.30 | 25 | 13 | 62 |
| H-1 | -5.58 | 7 | 16 | 77 | H-1 | -5.78 | 8 | 15 | 77 |
| H-2 | -5.97 | 31 | 11 | 58 | H-2 | -6.66 | 87 | 4 | 9 |
| H-3 | -7.13 | 65 | 3 | 32 | H-3 | -6.88 | 60 | 13 | 27 |
| H-4 | -7.21 | 70 | 1 | 28 | H-4 | -7.29 | 0 | 2 | 98 |
| H-5 | -7.28 | 19 | 41 | 40 | H-5 | -7.39 | 0 | 1 | 99 |

| **AzDT-5** | eV | bt* | HC=N | DAT |
|---|---|---|---|---|
| L+5 | -0.50 | 4 | 3 | 93 |
| L+4 | -0.72 | 33 | 11 | 56 |
| L+3 | -0.94 | 2 | 1 | 97 |
| L+2 | -1.02 | 13 | 3 | 84 |
| L+1 | -1.98 | 32 | 34 | 34 |
| LUMO | -2.77 | 43 | 27 | 30 |
| HOMO | -5.17 | 35 | 12 | 53 |
| H-1 | -5.65 | 12 | 15 | 74 |
| H-2 | -6.61 | 46 | 12 | 42 |
| H-3 | -7.30 | 80 | 0 | 20 |
| H-4 | -7.35 | 0 | 1 | 99 |
| H-5 | -7.36 | 0 | 1 | 99 |

* bPh – biphenyl; tt - thieno[3,2-b]thiophene; bt - 2,2'-bithiophene; DOT - 2,5-diamino-thiophene-3,4-dicarboxylic acid diethyl ester

Table S3. The calculated electronic transitions corresponding to excitation wavelength in $CHCl_3$ solution.

| | $\lambda_{exp}$ | $\lambda_{calc}$ ($f$)[#] | transition | character |
|---|---|---|---|---|
| **AzDT-1** | 401 | 410.0 (0.4245)<br>378.3 (0.3185) | HOMO→LUMO (23%); HOMO→L+1 (67%)<br>H-1→L+1 (89%) | $\pi_{DAT}\rightarrow\pi_{Ph\text{-}CH=N}^{*}$ |
| **AzDT-2** | 428 | 435.7 (0.0533) | H-1→LUMO (96%) | $\pi_{DAT}\rightarrow\pi_{bPh\text{-}CH=N}^{*}$ |
| **AzDT-3** | 445 | 482.8 (0.8738) | HOMO→LUMO (97%) | $\pi_{DAT}\rightarrow\pi^{*}$ |
| **AzDT-4** | 527 | 572.6 (0.9255)<br>468.8 (0.1252) | HOMO→LUMO (98%)<br>H-1→LUMO (95%); | $\pi_{DAT}\rightarrow\pi_{ttN=CH}^{*}$ |
| **AzDT-5** | 500 | 475.1 (0.112) | HOMO→L+1 (85%) | $\pi_{DAT}\rightarrow\pi^{*}$ |

[#] into account were taken the calculated transitions at the wavelength closest to the experimental data with the highest transition coefficient in this energy range

Table S4. Geometrical parameters of the imine molecules.

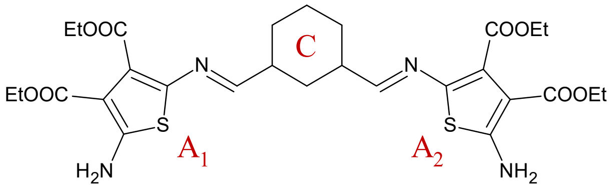


| | Dipole moment [D] | ∠ $A_1$-C [°] | ∠ $A_2$-C [°] |
|---|---|---|---|
| AzDT-1 | 8.32 | 2.29 | 32.00 |
| AzDT-2 | 2.96 | 2.37 | 37.74 |
| AzDT-3 | 9.94 | 34.55 | 55.03 |
| AzDT-4 | | | |
| $S_0$ | 6.58 | 0.88 | 31.07 |
| $S_1$ | 7.03 | 0.76 | 17.27 |
| $S_2$ | 6.84 | 1.44 | 27.10 |
| $T_1$ | 7.99 | 0.66 | 15.43 |
| $T_2$ | 6.84 | 1.44 | 27.10 |
| $T_3$ | 9.27 | 4.05 | 84.55 |
| AzDT-5 | | | |
| $S_0$ | 5.70 | 1.39 | 1.86 |
| $S_1$ | 5.79 | 1.69 | 3.18 |
| $S_2$ | 6.04 | 1.46 | 3.67 |
| $T_1$ | 5.71 | 0.99 | 4.64 |
| $T_2$ | 5.52 | 6.18 | 7.72 |
| $T_3$ | 6.08 | 2.15 | 2.91 |

## 12. UV-Vis spectra

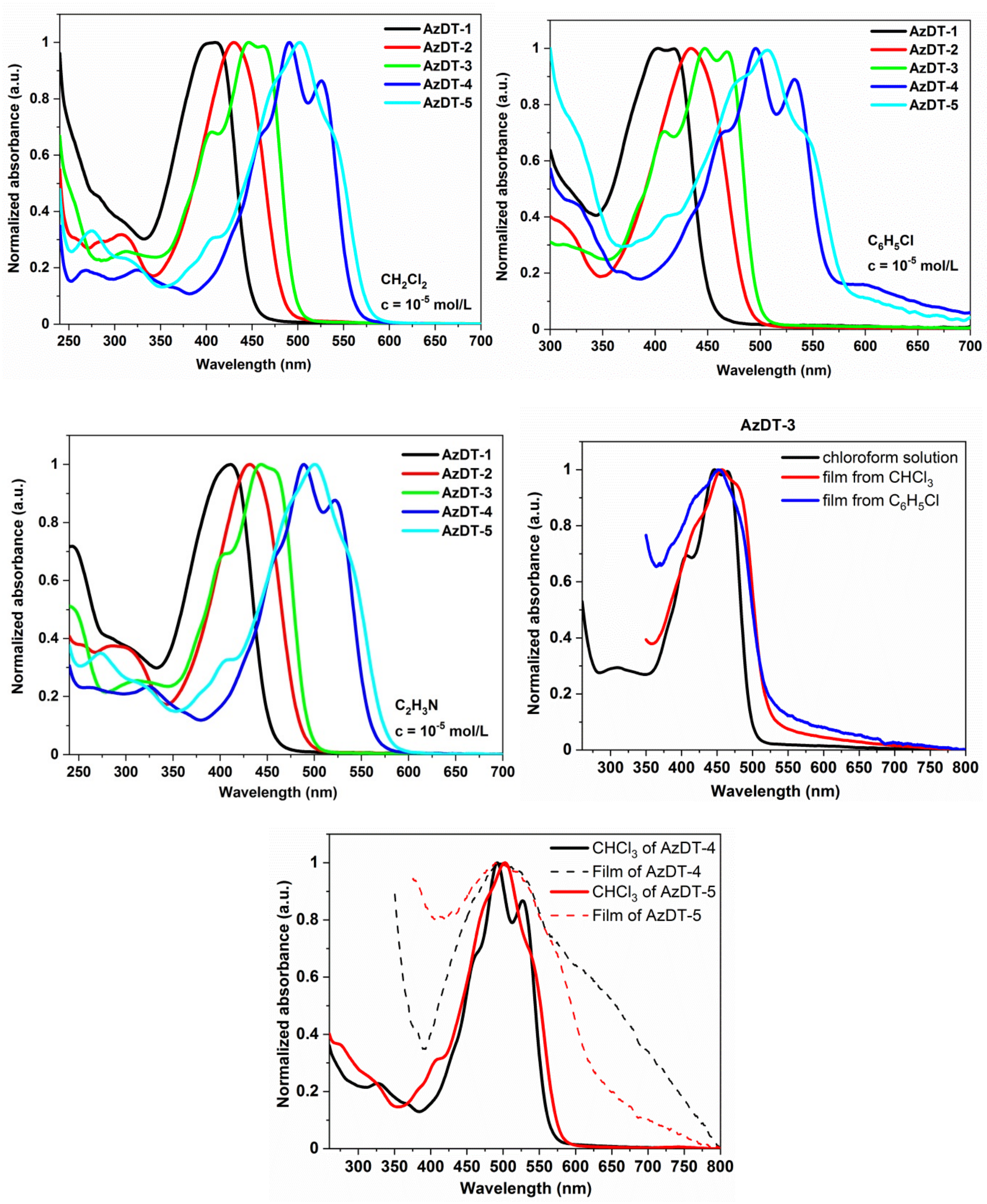


Fig. S9. The UV-Vis spectra of imino-bis-thiophenederivativesin solutions: dichloromethane, chlorobenzene and acetonitrile and in film compare with solutions.

## 13. PL spectra

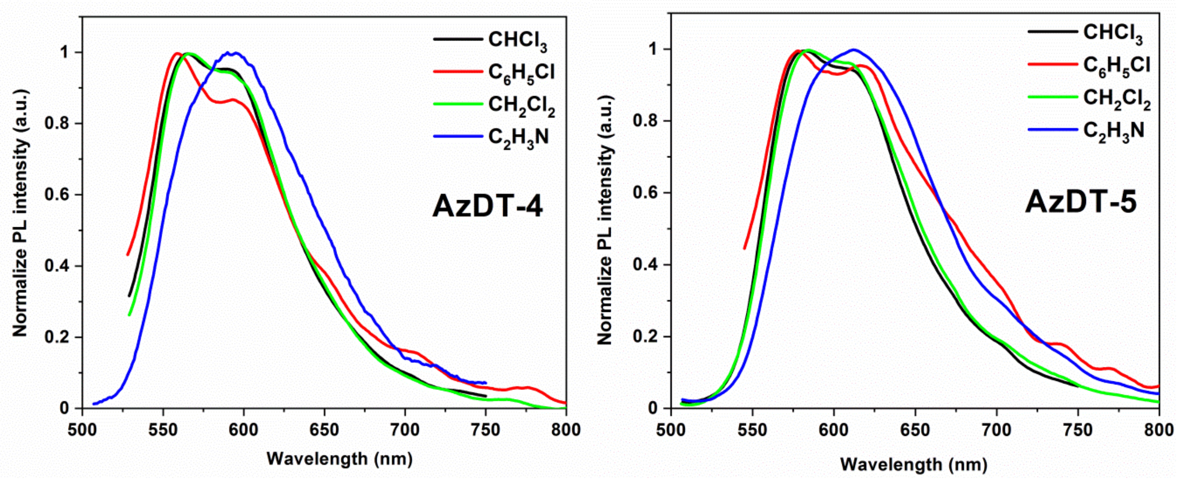


Fig. S10. The PL spectra of the AzDT-4 and AzDT-5 in four different solvents ($\lambda_{ex}$taken from the UV-Vis spectra, presented in Table 3 in the main manuscript).

## 14. The root-mean-square parameter

Table S5. The root-mean-square parameter.

| Device structure | RMS [nm] |
|---|---|
| FTO/b-$TiO_2$/m-$TiO_2$ | 20 |
| FTO/b-$TiO_2$/m-$TiO_2$/perowskit | 133 |
| FTO/b- $TiO_2$/m-$TiO_2$/perowskit/AzDT-1 | 85 |
| FTO/b- $TiO_2$/m-$TiO_2$/perowskit/AzDT-2 | 75 |
| FTO/b- $TiO_2$/m-$TiO_2$/perowskit/AzDT-3 | 85 |
| FTO/b- $TiO_2$/m-$TiO_2$/perowskit/AzDT-4 | 80 |
| FTO/b- $TiO_2$/m-$TiO_2$/perowskit/AzDT-5 | 90 |

RMS - The root-mean-square parametercharacterizing the surface roughness

## 15. SEM images

a)

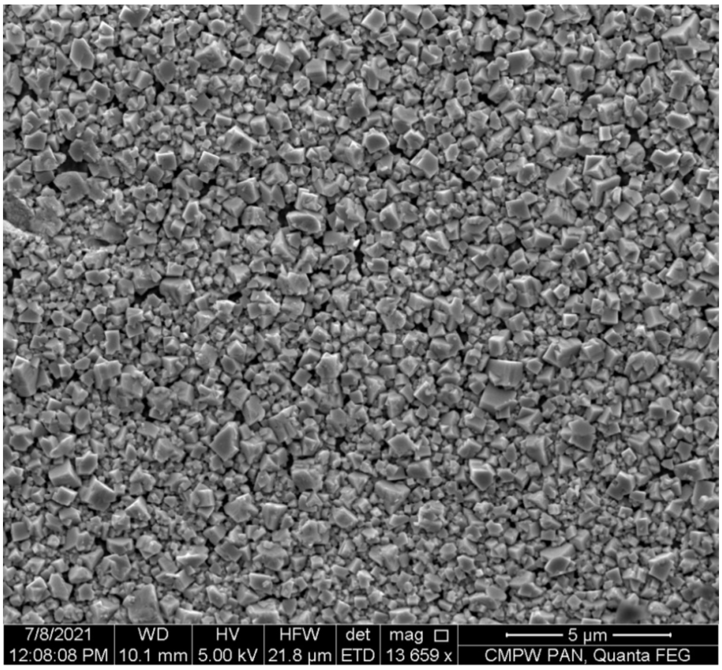


b)

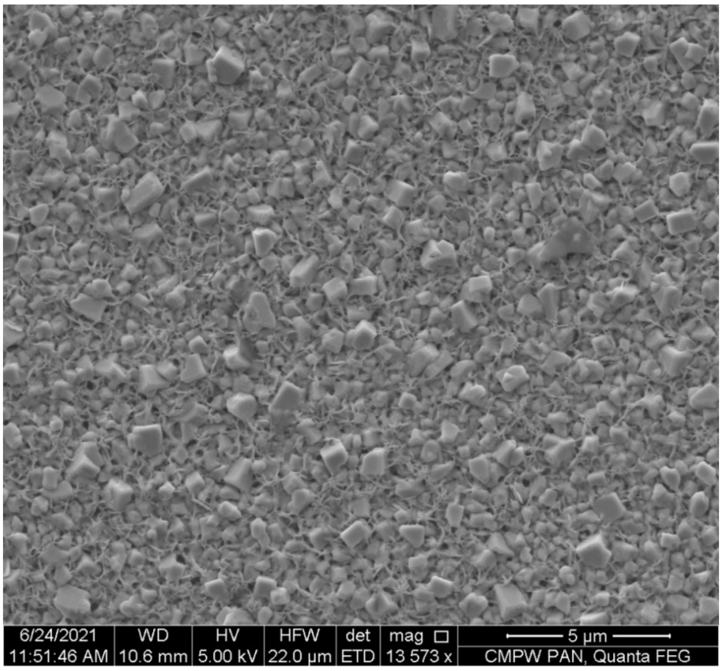


Fig. S11. a) SEM image of the perovskite layer and b) SEM image of the perovskite layer covered with AzDT-3, view from the top.

## 16. Solar cell data

Table S6. Photovoltaicproperties of the best fabricated hybrid perovskite solar cells: FTO/b-$TiO_2$/m-$TiO_2$/perovskite/Au, and $TiO_2$/perovskite/AzDTs:$V_{Li-TFSI}$ /Au.

| **AzDT** ($V_{Li-TFSI}$[μL]) | | $I_{sc}$ [mA] | $J_{sc}$ [mA/cm$^2$] | $V_{oc}$ [mV] | FF [-] | PCE [%] |
|---|---|---|---|---|---|---|
| - | The best | 2.31 | 9.24 | 156.10 | 0.25 | 0.41 |
| | Forward | 2.26 ± 0.05 | 9.04 ± 0.20 | 131 ± 25 | 0.25 ± 0.00 | 0.34 ± 0.07 |
| | Backward | 1.98 ± 0.02 | 7.92 ± 0.08 | 144.8 ± 7.4 | 0.25 ± 0.00 | 0.33 ± 0.02 |
| **spiro-MeOTAD** (17.50) | The best | 3.84 | 15.35 | 739.80 | 0.42 | 5.05 |
| | Forward | 3.83 ± 0.01 | 15.32 ± 0.03 | 744.66 ± 4.86 | 0.42±0.00 | 5.00 ± 0.05 |
| | Backward | 3.59 ± 0.04 | 14.35 ± 0.16 | 719.19± 0.03 | 0.35 ± 0.00 | 3.78 ± 0.03 |
| **AzDT-1** (8.75) | The best | 0.97 | 3.88 | 603.90 | 0.41 | 1.10 |
| | Forward | 0.98 ± 0.01 | 3.93 ± 0.05 | 518 ± 85 | 0.35 ± 0.06 | 0.81 ± 0.29 |
| | Backward | 0.73 ± 0.01 | 2.92 ± 0.05 | 521.3 ± 9.8 | 0.33 ± 0.00 | 0.58 ± 0.02 |
| **AzDT-1** (17.50) | The best | 0.92 | 3.68 | 425.70 | 0.33 | 0.59 |
| | Forward | 0.93 ± 0.01 | 3.71 ± 0.03 | 372 ± 54 | 0.31 ± 0.02 | 0.49 ± 0.10 |
| | Backward | 0.60 ± 0.01 | 2.41 ± 0.06 | 335 ± 15 | 0.31 ± 0.01 | 0.29 ± 0.02 |
| **AzDT-1** (35.00) | The best | 1.68 | 6.72 | 530.30 | 0.34 | 1.37 |
| | Forward | 1.65 ± 0.03 | 6.60 ± 0.14 | 519 ± 12 | 0.34 ± 0.00 | 1.32 ± 0.05 |
| | Backward | 1.26 ± 0.00 | 5.04 ± 0.01 | 537.3 ± 7.4 | 0.35 ± 0.01 | 1.07 ± 0.04 |
| **AzDT-2** (8.75) | The best | 1.90 | 7.60 | 760.80 | 0.34 | 2.24 |
| | Forward | 1.89 ± 0.01 | 7.57 ± 0.04 | 736 ± 25 | 0.33 ± 0.01 | 2.09 ± 0.15 |
| | Backward | 1.95 ± 0.01 | 7.81 ± 0.05 | 635.3 ± 0.4 | 0.18 ± 0.01 | 1.04 ± 0.02 |
| **AzDT-2** (17.50) | The best | 1.63 | 6.52 | 759.50 | 0.42 | 2.38 |
| | Forward | 1.61 ± 0.03 | 6.43 ± 0.10 | 756.3 ± 3.2 | 0.42 ± 0.00 | 2.34 ± 0.05 |
| | Backward | 1.50 ± 0.03 | 6.02 ± 0.13 | 713 ± 15 | 0.30 ± 0.01 | 1.45 ± 0.09 |
| **AzDT-2** (35.00) | The best | 0.98 | 3.92 | 301.10 | 0.31 | 0.42 |
| | Forward | 0.98 ± 0.00 | 3.90 ± 0.02 | 185 ± 116 | 0.30 ± 0.01 | 0.26 ± 0.16 |
| | Backward | 0.78 ± 0.02 | 3.12 ± 0.10 | 100 ± 23 | 0.28 ± 0.02 | 0.10 ± 0.04 |
| **AzDT-3** (8.75) | The best | 3.38 | 13.50 | 673.50 | 0.35 | 3.64 |
| | Forward | 3.38 ± 0.00 | 13.52 ± 0.02 | 668± 60 | 0.35 ± 0.00 | 3.58 ± 0.06 |
| | Backward | 3.42 ± 0.03 | 13.68 ± 0.12 | 609± 17 | 0.27 ± 0.00 | 2.61 ± 0.10 |
| **AzDT-3** (17.50) | The best | 1.95 | 7.80 | 680.50 | 0.35 | 2.11 |
| | Forward | 1.91 ± 0.04 | 7.63 ± 0.17 | 584± 97 | 0.36 ± 0.01 | 1.84 ± 0.27 |
| | Backward | 1.49 ± 0.11 | 5.95 ± 0.45 | 615± 46 | 0.26 ± 0.01 | 1.08 ± 0.13 |
| **AzDT-3** (35.00) | The best | 0.70 | 2.80 | 513.90 | 0.35 | 0.57 |
| | Forward | 0.59 ± 0.11 | 2.36 ± 0.44 | 314 ± 200 | 0.33 ± 0.02 | 0.30 ± 0.27 |
| | Backward | 0.34 ± 0.02 | 1.37 ± 0.08 | 220 ± 52 | 0.29 ± 0.00 | 0.10 ± 0.03 |
| **AzDT-4** (8.75) | The best | 0.98 | 3.92 | 708.10 | 0.46 | 1.46 |
| | Forward | 0.88 ± 0.10 | 3.50 ± 0.42 | 710.8 ± 2.7 | 0.46 ± 0.00 | 1.30 ± 0.16 |
| | Backward | 0.43 ± 0.01 | 1.71 ± 0.03 | 653.0 ± 4.5 | 0.38 ± 0.00 | 0.48 ± 0.02 |
| **AzDT-4** (17.50) | The best | 0.92 | 3.68 | 758.60 | 0.59 | 1.88 |
| | Forward | 0.81 ± 0.11 | 3.24 ± 0.44 | 753.5 ± 5.1 | 0.55 ± 0.04 | 1.55 ± 0.33 |
| | Backward | 0.45 ± 0.03 | 1.82 ± 0.10 | 507 ± 11 | 0.32 ± 0.00 | 0.33 ± 0.04 |
| **AzDT-4** (35.00) | The best | 1.20 | 4.80 | 778.90 | 0.50 | 2.14 |
| | Forward | 1.08 ± 0.12 | 4.32 ± 0.48 | 774.0 ± 4.9 | 0.48 ± 0.02 | 1.85 ± 0.29 |
| | Backward | 0.72 ± 0.02 | 2.86 ± 0.09 | 688.1 ± 8.4 | 0.27 ± 0.00 | 0.60 ± 0.04 |
| **AzDT-5** (8.75) | The best | 1.25 | 5.00 | 798.00 | 0.45 | 2.04 |
| | Forward | 1.14 ± 0.11 | 4.56 ± 0.44 | 796.5 ± 1.6 | 0.45 ± 0.00 | 1.85 ± 0.19 |
| | Backward | 0.78 ± 0.00 | 3.12 ± 0.00 | 733.8 ± 5.1 | 0.32 ± 0.01 | 0.85 ± 0.02 |
| **AzDT-5** | The best | 0.82 | 3.28 | 753.40 | 0.41 | 1.16 |

| | | | | | | |
|---|---|---|---|---|---|---|
| (17.50) | Forward | 0.78 ± 0.04 | 3.13 ± 0.16 | 730 ± 23 | 0.38 ± 0.03 | 1.01 ± 0.15 |
| | Backward | 0.59 ± 0.00 | 2.37 ± 0.01 | 664 ± 30 | 0.35 ± 0.01 | 0.63 ± 0.03 |
| **AzDT-5** (35.00) | The best | 0.43 | 1.72 | 640.30 | 0.33 | 0.42 |
| | Forward | 0.40 ± 0.03 | 1.61 ± 0.12 | 544 ± 96 | 0.31 ± 0.02 | 0.32 ± 0.11 |
| | Backward | 0.38 ± 0.00 | 1.53 ± 0.01 | 471.1 ± 3.4 | 0.29 ± 0.00 | 0.24 ±0.01 |

$V_{Li\text{-}TFSI}$ - volume of additive Li-TFSI solution.

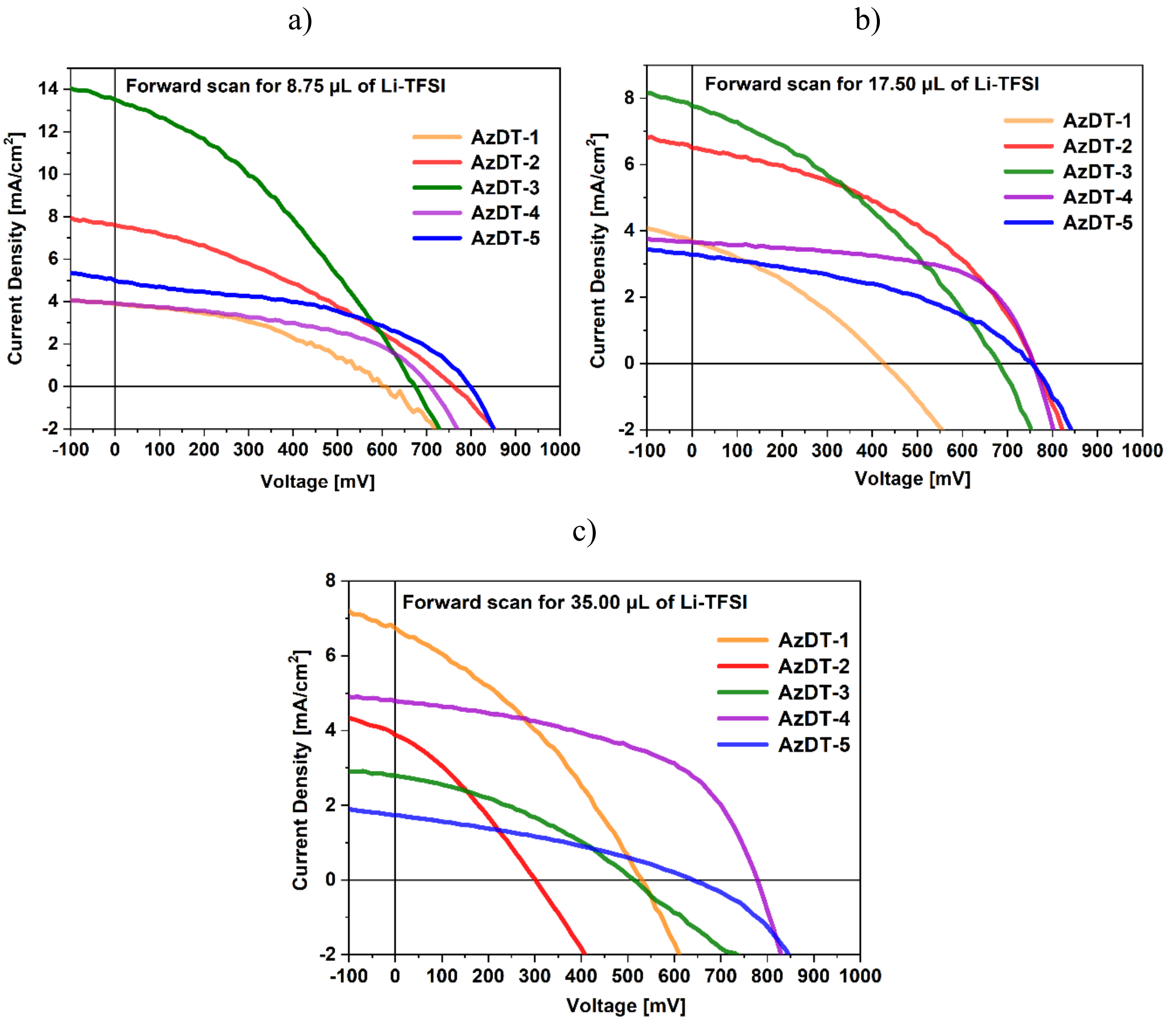


Fig. S12.The current-voltage characteristics for selected devices for a) 8.75µL, b) 17.50 µL and c) 35.00 µL of Li-TFSI dopant.

## 17. Density and dielectric constant

Table S6. Density anddielectric constant.

| Code | Density[a] [g/cm$^3$] | Dielectric constant [-] [b] B3lyp/6-311+G(d,p) |
|---|---|---|
| AzDT-1 | 1.39 | 3.17 |
| AzDT-2 | 1.41 | 4.61 |
| AzDT-3 | 1.32 | 4.31 |
| AzDT-4 | 1.45 | 5.37 |
| AzDT-5 | 1.44 | 6.52 |

[a] The absolute density was measured using a helium pycnometerAccuPyc 1330 Micrometrics.
[b] Dielectric constant calculated on the basis of experimentally determined density.

## 18. OFET current-voltage characteristics

a)

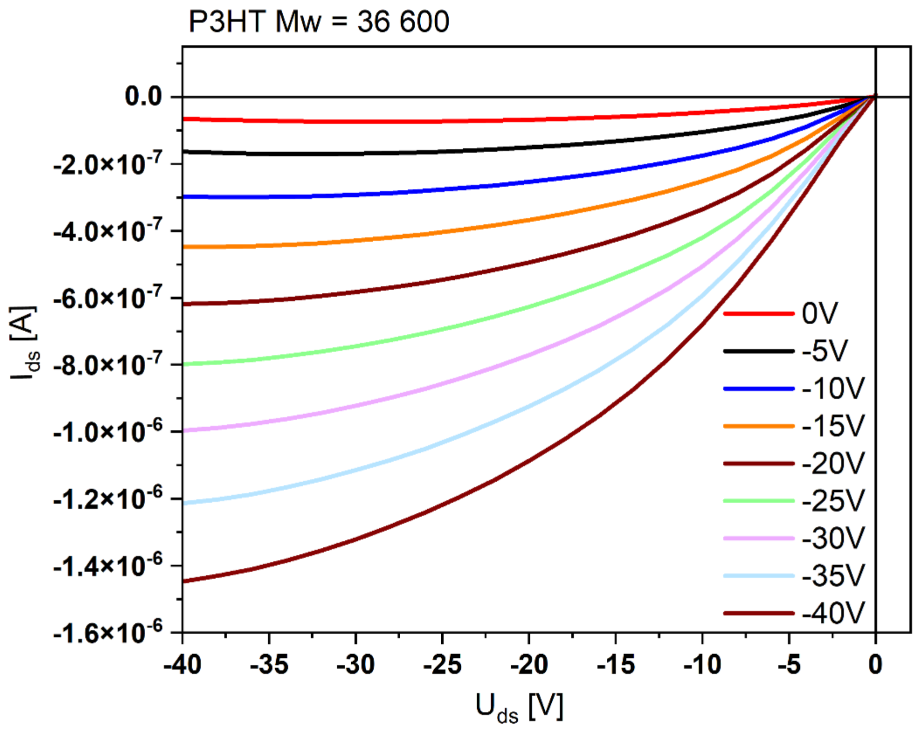

b)

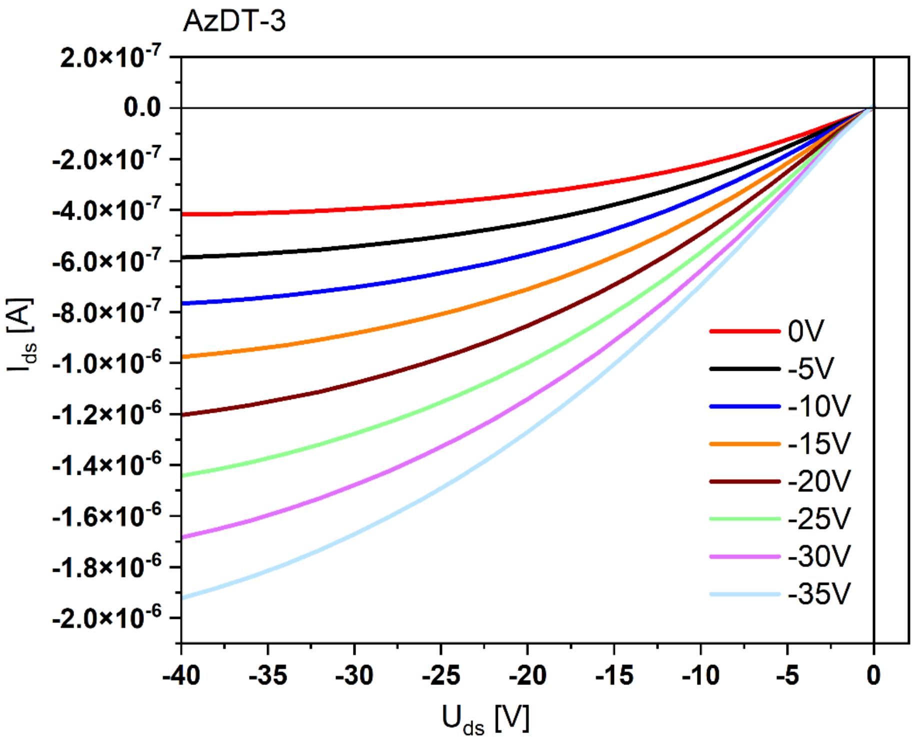


Fig. S13. $I_{ds}$ – $U_{ds}$ characteristics of FET device with a) P3HT active layer and b) AzDT-3.
